# The Absoluteness of Semantic Processing: Lessons from the Analysis of Temporal Clusters in Phonemic Verbal Fluency

**DOI:** 10.1371/journal.pone.0115846

**Published:** 2014-12-23

**Authors:** Isabelle Vonberg, Felicitas Ehlen, Ortwin Fromm, Fabian Klostermann

**Affiliations:** Charité – University Medicine Berlin, Department of Neurology, CBF, Motor and Cognition Group, Berlin, Germany; Beijing Normal University, China

## Abstract

**Background:**

For word production, we may consciously pursue semantic or phonological search strategies, but it is uncertain whether we can retrieve the different aspects of lexical information independently from each other. We therefore studied the spread of semantic information into words produced under exclusively phonemic task demands.

**Methods:**

42 subjects participated in a letter verbal fluency task, demanding the production of as many *s*-words as possible in two minutes. Based on curve fittings for the time courses of word production, output spurts (temporal clusters) considered to reflect rapid lexical retrieval based on automatic activation spread, were identified. Semantic and phonemic word relatedness within versus between these clusters was assessed by respective scores (0 meaning no relation, 4 maximum relation).

**Results:**

Subjects produced 27.5 (±9.4) words belonging to 6.7 (±2.4) clusters. Both phonemically and semantically words were more related within clusters than between clusters (phon: 0.33±0.22 vs. 0.19±0.17, *p*<.01; sem: 0.65±0.29 vs. 0.37±0.29, *p*<.01). Whereas the extent of phonemic relatedness correlated with high task performance, the contrary was the case for the extent of semantic relatedness.

**Conclusion:**

The results indicate that semantic information spread occurs, even if the consciously pursued word search strategy is purely phonological. This, together with the negative correlation between semantic relatedness and verbal output suits the idea of a semantic default mode of lexical search, acting against rapid task performance in the given scenario of phonemic verbal fluency. The simultaneity of enhanced semantic and phonemic word relatedness within the same temporal cluster boundaries suggests an interaction between content and sound-related information whenever a new semantic field has been opened.

## Introduction

Lexical search can imply different selection strategies. Certainly, we mostly choose words according to the content we aim to convey [1], [2], but other criteria may prevail under specific demands, for example, when testing ‘letter’ verbal fluency (VF). In respective tasks, phonemic VF is assessed on condition of producing as many distinct words as possible beginning with a defined letter per time unit. In this context, it is an interesting question to which extent we can control the search mode, adapt it to the task-inherent demand, and scan the mental lexicon only under the premise of phonological word features, leaving semantic information aside. How this is answered depends on whether one conceives the processing of sound and content-related word features as necessarily interwoven or potentially separate, and which automaticity one attributes to the respective operations.

In this regard, it is a widespread view that in ‘lexical networks’ data about word meaning and word sound form representation ‘nodes’ and that the internodal connectivity correlates with the similarity of the information stored therein [3]. In this architecture, the activation of a specific piece of lexical information automatically primes data stored in associated nodes [3]–[6], facilitating word availability. Support for this view came from the analysis of word production dynamics in VF and recall tasks, e. g. on the basis of curve fitting approaches, which allow to model the time course of word production [7]–[11]. Next to approximating the overall output process, this also provides a criterion for the identification of so called ‘temporal clusters’, defined as sequences of words produced at a higher than mathematically predicted rate [10], [12]–[15]. Thus defined clusters are thought to reflect the rapid recruitment of word candidates primed by automatic activation spread within a semantic field, whereas the deceleration between subsequent clusters rather indicates the slower retrieval of less associated lexical concepts, already belonging to another semantic field [10], [16].

In line with this concept, it could indeed be shown that content relations are higher amongst words within than between temporal clusters in semantic VF tasks [10], [14], [15]. An equivalent study for letter VF has not been performed so far. This could certainly provide interesting data with respect to the current research question; first, because the variation of phonemic word relation between versus within temporal clusters in letter VF might shed light on whether the organization principles presumed for semantic processing are also relevant in the lexico-phonological domain; and, second, since it could help to determine if semantic field information influences letter VF, although the latter does not require a conscious content-based search strategy [16]–[24].

In this regard, different theoretical scenarios might be taken into account. First, the relatedness of words might be invariant within versus between temporal clusters in letter VF, be it phonemic or semantic. In this case, for phonological word search other principles than the ‘automatic field activation’, thought to prevail in semantic word search, would have to be considered. Second, only the phonemic relatedness of words could rise up within as compared to between clusters, implying automatic activation of sound without concomitant content-related information, and compatible with the possibility of actively controlling basic lexical search modes. Finally, just as in semantic VF, words produced in letter VF tasks might be stronger semantically related within than between temporal clusters, indicating automatic spread of content-based information into the phonological word production process and in line with the idea of a semantic ‘default mode’, as proposed by influential word production models [4], [5], [25]–[27]. Whether this would coincide with phonological clustering should further reveal the interactivity of automatic semantic and phonological word processing.

To differentiate between these alternative hypotheses, we performed a temporal cluster analysis of words produced in the German standard task for letter VF in 42 healthy native speakers. The semantic relatedness of the produced words was rated by a separate group of participants, whilst the assessment of phonemic relatedness followed the widely used conventions introduced by Troyer et al. [16]. The findings are discussed in the context of relevant concepts of lexical processing [28].

## Methods

### Participants

42 subjects free of neurological and psychiatric diseases (no symptoms according to the AMDP score, [29]) and not taking any centrally acting drugs participated in the study (18 female/24 male, age 49.9±19.7). They had a mean school education of 11.7±1.7 years. All participants were native German speakers. They gave written informed consent to the study protocol approved by the Ethics Committee of the Charité (protocol number EA2/047/10).

### Procedure

Participants performed the phonemic task of the standard German VF test (‘Regensburger Wortflüssigkeits-Test’) [30], requiring to name as many words as possible starting with the letter “s” within 2 minutes. The output was digitally recorded with the audio software Audacity (operating software Windows).

The following scores were obtained: total number of words produced, number of temporal clusters, cluster size, number of switches, within-cluster pauses and between-cluster pauses. Patients should avoid to name proper words, or repeat words stems. However, since such utterances are considered as informative about underlying cognitive processes [16], they were included in the analysis. Meta-comments (e. g., “I don't know any more words” or “I think I already mentioned that word”) were excluded.

The assumption of temporal clusters and switches was based on the deviation from the mathematically predicted intervals between the words produced during the phonemic VF task, based on curve fitting (for details see below).

### Analysis of Clusters and Switches

The first steps for the assessment of the verbal output dynamics were the determination of the points in time at which words were produced, the pause lengths, and the word durations at a temporal resolution of 1 ms (Audacity). Afterwards, the time course of word production was modeled individually by curve fitting of the verbal output applying exponential functions. This approach was chosen because it has been previously shown to provide a good approximation of the word production dynamics in VF and recall experiments [7], [11], [31]–[34].

For the curve fitting, the total number of words produced in the phonemic task N(t) was calculated as N(t)  =  G (1-e^αt + β^), where G represents the volume of the resource (words available for production). Because of N(0)  = −G α e^β^, the parameters α and β implicitly describe the slope of the curve in t_(0)_. By taking the logarithm Λ(t)  =  ln (1−N(t)/G)  = α t+β, this function was linearized and the variation of the results could be subjected to a subsequent least-mean-square analysis. Thus, in an iterative procedure the exponential function fitting the individual verbal output dynamics best was identified.

Based on the best fitting function per subject, clusters and switches were analyzed according to the slope-difference algorithm by Gruenewald and Lockhead [10] (also see [12]).

The algorithm is based on the difference between the actual time intervals between the words and the time intervals predicted by the best fitting function. If the actual slope between two consecutive words was steeper than predicted, the two words were considered to belong to a cluster. If the actual slope was lower, they were regarded to be part of different clusters.

For the analysis of the size of the temporal clusters and the number of switches between these clusters, we followed the common conventions of Troyer et al. [16]. Cluster size was counted beginning with the second word in a cluster (i.e., a two-word cluster was given a size of 1, a three-word cluster a size of 2, etc.). Switches were counted as the number of transitions between the clusters, including single words.

### Ascertainment of semantic relatedness

For the analysis of the semantic relatedness between the words produced in the VF task, a separate group of 30 participants (20 female/10 male; age 50.3±17.92; years of school education: 12.13±1.48; not statistically different from the VF participants, *p* = .258; all native German speakers) rated how close the meaning of consecutive words was, using a scale from 0 to 4 (0 =  no semantic relation; 1 =  weak semantic relation; 2 =  moderate semantic relation; 3 =  strong semantic relation; 4 =  very high semantic relation). For the scoring, they were provided with the lists of all words produced in the VF task per subject. Their instruction was to rate the semantic relatedness between the successive words in the same order in which they were generated. In so doing, the purpose of the study and the boundaries of the determined temporal clusters remained unrevealed to them.

### Ascertainment of phonemic relatedness

The phonemic relatedness between the words produced in the letter VF task was determined based on the method introduced by Troyer et al. [16]. Accordingly, successively generated words were considered phonemically related when a) they started with the same two initial phonemes (e.g., *Sp*ort, *Sp*iel), b) they differed only in a vowel sound (e.g., S*ü*den, s*ie*den), c) they rhymed (e.g., Schwein, Stein), or d) they were homophones, as indicated by the participant (e.g., *Stiel, Stil*).

Since the VF task required the naming of words starting with the same letter, the most basic phonemic relation between generated words was their alliteration. Since all words, except for erroneous ones, were thus alliterated, alliterations were given the base value 0 on a phonemic relatedness score from 0 to 4 (0 =  alliteration; 1 =  two initial phonemes identical; 2 =  different vowel only; 3 =  rhyme; 4 =  homophone).

### Statistical Analysis

To find out whether phonemic and semantic relatedness differed within versus between clusters, we determined the mean relatedness scores for the words belonging to these VF sections for each participant, on the basis of the ratings described above. For comparing the respective rating scores, the non-parametric Wilcoxon signed-rank test for paired samples was used.

Further, Spearman's correlations were calculated between the individual number of words and the slope of the respective exponential curve at t_(0)_. Additionally, correlations were determined on the one hand between the number of words and the number of clusters, cluster size and the number of switches, and on the other hand between phonemic/semantic relatedness and switching times. Finally, it was tested whether semantic and phonemic relatedness scores covaried and if either score correlated with the number of words produced, both for all words, whether within or between clusters, and for the words within clusters.

All statistical tests were performed with SPSS version 19.

## Results

Modelling the 42 VF datasets by the exponential function, as described above, the method of least squares yielded a mean sigma of the curve-fit of 0.65±0.2. The curve's slope at t_(0)_ was 20.79±8.38 and it correlated with the number of words produced (r = 0.578; *p* = .01).

### Clusters and Switches

The results of the cluster analysis as well as the mean number of words are summarised in *Table 1*. The average pause duration between two consecutive words within a cluster and that between clusters was 2.29±1.09 s and, respectively, 7.35±4.32 s. The Wilcoxon signed rank test showed a significant difference between these durations (*p*<.001).

**Table 1:** Cluster and switching results.

	Mean	SD
Number of words	27.5	9.40
Number of clusters	6.74	2.41
Cluster size	2.72	1.06
Number of switches	10.55	4.06

The number of words correlated with the number of clusters (r = .776; *p* = .000) as well as with the number of switches (r = .87; *p* = .000). Cluster size and number of words did not correlate with each other (r = .059; *p* = .71). A negative correlation was given between the number of clusters and cluster size (r = −.509; *p* = .001).

### Semantic and phonemic relatedness

The ratings of the semantic and phonemic relatedness are summarised in *Table 2*.

**Table 2:** Relatedness scores between and within clusters.

Scores	Mean	SD
Semantic relatedness within clusters	0.65	0.29
Semantic relatedness between clusters	0.37	0.29
Phonemic relatedness within clusters	0.33	0.22
Phonemic relatedness between clusters	0.19	0.17

For the comparison of the semantic and phonemic relatedness scores for words within versus between clusters, significant differences were identified (semantic relatedness: *p*<.001; phonemic relatedness: *p*<.001).

A low, but significant correlation between phonemic relatedness within and between clusters was found (r = .38; *p* = .013), i. e., the higher phonemic relatedness was within clusters, the higher it tended to be between them. No such correlation was found for semantic relatedness (r = .271; *p* = .083). Further, there was a moderate correlation between overall phonemic relatedness and switching time, i. e, the interval needed for cluster transitions, (r = − 0.41; *p* = .008), which, in turn, correlated positively to semantic relatedness (r = 0.39; *p* = .01).

### Effect of relatedness on the number of words produced

To investigate the relationship between word relatedness and verbal output a correlation analysis was performed between both intra-cluster relatedness scores as well as overall relatedness scores (i.e. of words within and between clusters together) and the number of words.

A significant correlation was found between intra-cluster and overall phonemic relatedness and the number of words produced (intra-cluster: r = .414; *p* = .006; overall: r = .452; *p* = .003). A weak negative correlation was found between the number of words produced and overall semantic relatedness (r = −.323; *p* = .037), similarly, but slightly below the level of statistical significance the r-value for the correlation between the number of words produced and the intra-cluster semantic relatedness was r = −.294; *p* = .058.

No correlation was obtained between overall or intra-cluster phonemic and semantic relatedness (overall: r = −.186; *p* = .239; intra-cluster: r = −.171; *p* = .278).

### Correlation between word relatedness and cluster behaviour

There was no correlation between the number of clusters and overall semantic relatedness. But there was a correlation between the number of clusters and the words' overall phonemic relatedness (r = .505; *p* = .001). Cluster size did neither correlate with semantic nor with phonemic relatedness.

## Discussion

In this study, temporal clusters in a phonemic VF task were assessed as sequences of words produced above the mathematically predicted rate. Within the thus defined temporal clusters, the relatedness of words was higher than between them, both in phonemic and semantic terms. Furthermore, while the number of the words produced correlated positively with their phonemic relatedness, the contrary was the case with respect to semantic relatedness.

The higher phonemic relatedness of words within than between temporal clusters is compatible with results of previous investigations of phonemic VF [16]–[18], [21], [35], [36] in which clusters were, however, defined based on a-priori or a-posteriori of definitions of word relatedness without consideration of VF dynamics. Unlike in these studies, we here used a completely data-driven approach defining clusters ‘temporally’ as word production spurts regardless of lexical relatedness. Comparable investigations have so far only been conducted for semantic VF, demonstrating higher within- than between-cluster relatedness of word meaning [8], [10], [14], [15]. This has been interpreted to reflect automatic activation spread across densely related ‘representation nodes’ within a given semantic field, facilitating fast verbal output and changing with slower cluster transitions whenever a field scan has been completed [3], [10], [11]. In analogy to this, the present results of higher phonemic within- than between-cluster relatedness is well compatible with a similar phonological network organisation and word processing therein.

However, the central finding of the current study was that although the participants were only asked to utter words beginning with the same letter, the temporal clusters produced were characterised by enhanced phonemic and, at the same time, semantic relatedness, a result that is supported by studies [37], [38] which described semantic effects in letter fluency tasks, without focussing on temporal aspects. A temporal co-occurrence of both semantic and phonemic word relatedness in production spurts has so far not been investigated. Our result thus relates to the question of how semantic and phonological processing steps may interact in word search, an issue controversially discussed [27], [28], [39]. Mostly, it is assumed that lexicalisation comprises two levels: the retrieval of semantic information and, second, of the word form [4], [5], [25], [26], [40]–[42]. Whereas ‘discrete’ models posit that semantic and phonological operations are serial and separate processes [5], [43]–[47], ‘interactive’ concepts assume that they overlap and influence each other [4], [26], [48]–[52]. The current findings are suggestive of the latter idea, since otherwise it would be difficult to explain how phonemic and semantic word characteristics could coincide within the same cluster boundaries. Beyond this notion, the obtained correlational findings might provide some additional insights into how this interaction might take place.

### High semantic relatedness – low word count

The inverse correlation between enhanced semantic relatedness and word count indicates that content search acts as an obstacle for phonemic VF. An explanation for this could be that – in line with prevailing ideas – the scanning of lexical contents is the fundamental working mode of the system [1], [2], [53]–[55]. A search based on semantic representations has, however, an intrinsically low probability to generate suitable word candidates in a phonemic VF task, since it would be incidental if activated semantic concepts also met the phonemic task criterion (with a likelihood equivalent to the percentage with which words with the demanded initial consonant ‘s’ are represented in the vocabulary of a participant). Thus, from the perspective of processing economy, target-oriented phonological scanning would certainly be advantageous. The finding of phonemic clustering in this study – indicating automatic activation spread of sound-based lexical information – and the positive correlation between phonemic relatedness and word count, indeed indicate an active phonemic search mode.

### High phonemic relatedness – high word count

As detailed above, for reasons of task-specificity, a parallel phonological search stream should increase the hit rate of activated word forms compared to the semantic scanning mode. Further, this process can be conceived to occur on a similar basis as proposed for semantic search [3]. The result of a relatively high phonemic word similarity within clusters which returned to lower levels between them suggests sequential phonological activation spread, resulting in consecutive intervals of rapid word production.

Having said this, it remains to be settled how phonological and semantic word scanning could effectuate the *same* production clusters. Without the presumption of specific interactions between parallel semantic and phonological search streams, this would be a highly unlikely situation, because there is no reason to presume that phonological word fields [56] are congruent with semantic fields.

### High phonemic and semantic word similarity within versus between clusters

**Figure 1:**
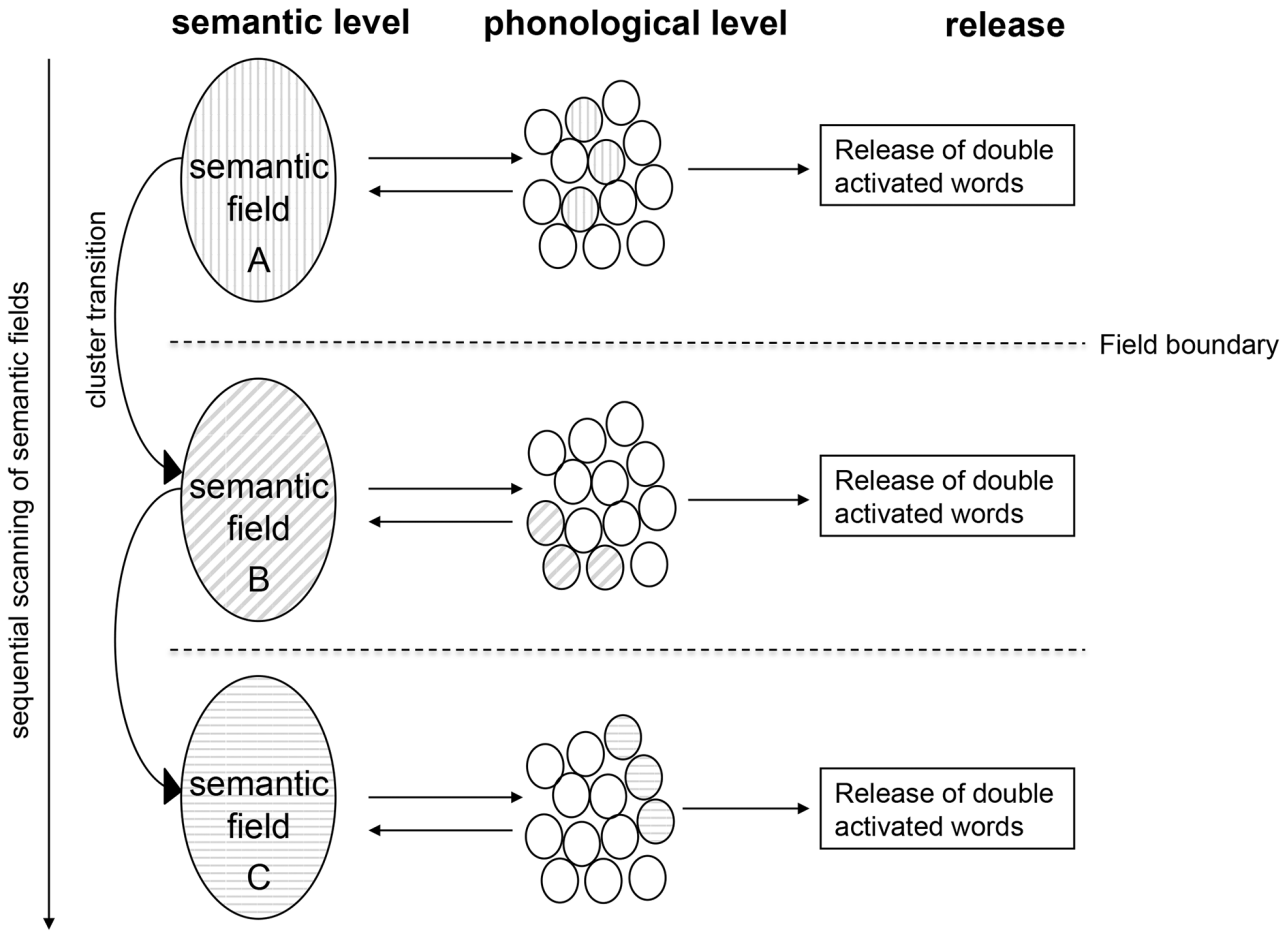
Cascaded lexical search model. Clusters are thought to reflect the cascaded processing of semantic and phonological information: In this view, word search originates from the activation of semantic fields (A, B, C). During respective field scans a parallel phonological search stream is activated. In letter VF, words would be released if a semantic concept can be aligned with a suitable phonological representation. A cluster transition occurs upon completion of these automatic and therefore rapid operations per field, when the next semantic category has to be accessed and phonemic alignment is restarted.

**Figure 2:**
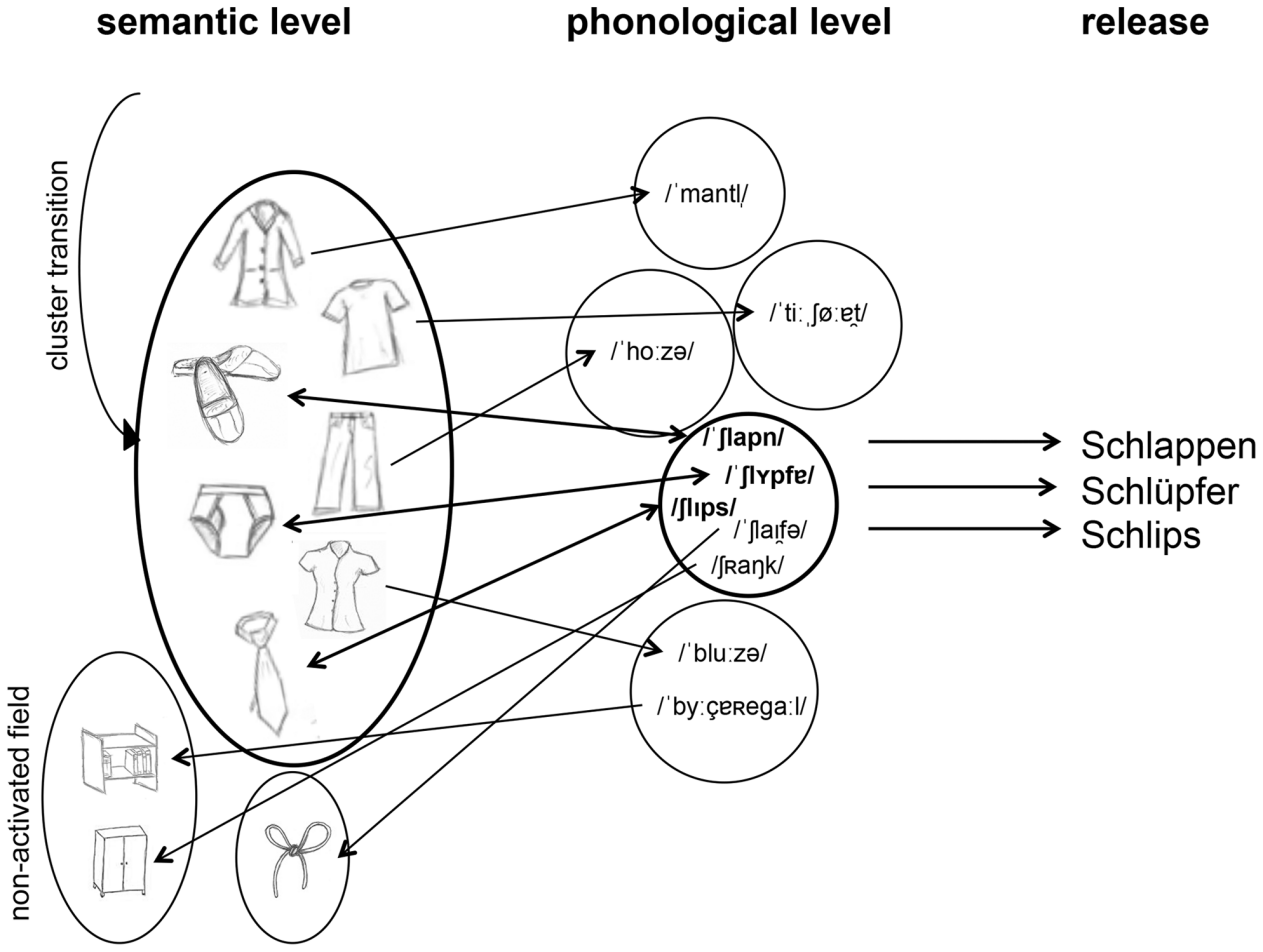
Model for enhanced semantic and phonemic word relatedness within clusters. After opening a semantic field (bold line ellipse, here for “clothes”), related concepts are automatically activated. The retrieved information activates phonological representations mostly unsuited for the ongoing requirement (initial letter *s*), but sometimes matching the letter VF demand (here for the first time when activating the concept for ‘Schlappen’, in German colloquial for slippers). On the other hand, information from the parallel phonological processing stream (bold line ellipse, here activating word forms with the same two initial phonemes/∫/and/l/) is aligned to concepts either within the set semantic field or outside of it (outside, for example, at activation of ‘Schleife’, meaning bow). Those word forms phonologically suitable and semantically preactivated have the best chance to be released, explaining the increase of phonemic and semantic word relations within temporal clusters in the verbal output.

How parallel search streams could lead to clusters with enhanced phonemic and semantic word relatedness shall be explained based on a model, sketched in Figs. 1 and 2. The presented view is compatible with interactive concepts of word production [4], [26], [51] and based on the idea that semantic and phonological activations spread in parallel with bidirectional information exchange, and on the widespread assumption that semantic concepts are activated first [5], [25], [26]. It further assumes that in the semantic-to-phonologic alignment process mainly ‘double-activated’ concepts are recruited for production, i. e. those word forms which are both semantically and phonologically similar among each other.

Within this framework, the identified increase of semantic and phonemic word relatedness in the same temporal clusters in letter VF could be explained as a combination of cascaded, interactive and automatic lexical processing. As a starting point, it is conceived that activation spreads through a given semantic field. Thus recruited semantic concepts activate the corresponding phonological word forms, mostly belonging to distinct phonological fields (according to the low probability that related semantic concepts correspond to phonemically related words). Further, it is assumed that the initial phonological recruitment initiates the automatic activation of phonologically related word forms. If, in a feedback process with the semantic level, one of the activated phonological forms corresponds to a concept belonging to the initially activated semantic field, the resulting double activation on both levels of lexical processing should facilitate the release of the matched word candidate for production – in contrast to phonological word forms which cannot be aligned with an available semantic concept.

The oscillation of semantic and phonemic word relatedness with repeated decreases between and increases within temporal clusters indicates that the described process sequence evolves iteratively, i. e., it is re-started on both levels once a new semantic field has been opened. Having said that, the moderate correlation of within- and between-cluster phonemic word relatedness found in the study suggests that the restarts of phonological activation spread do not occur completely at random, but in some vicinity to where the phonological process has led to before.

### Previous studies

Bidirectional exchange of semantic and phonological information and the idea of parallel phonological and semantic processing have been doubted [43], [57]. For example, Schriefers et al. [57] found that in a picture naming task the critical phase for the facilitation of target word production was earlier for semantically than for phonemically related prime words, suggestive of discrete, ‘semantic first’ lexical processing. On a similar note, Levelt et al. [43] investigated phonological-to-semantic information exchange based on the concept of ‘mediated priming’ (meaning that, e. g., the word *goal* would activate the word *sheep* via the primary activation of *goat*). The absence of respective effects was considered as an argument that phonological information would not influence semantic processing, reminiscent of discrete rather than interactive models. In other naming tasks, however, phonological activation of non-selected semantic information was found [51], [58]–[60].

Yet, the theorem of a discrete information flow from the semantic to the phonological level has been challenged by results on the occurrence of “mixed speech errors” [48], [61], [62]. In these production mistakes, an utterance is both semantically and phonemically related to an actually intended word (e.g. when a speaker articulates “rat” instead of “cat”). This effect occurs above chance probability for combined semantic and phonemic relations, and has been viewed as indicative of bidirectional information flow between both lexical processing levels [27], [48], [63]. Specifically, it was posited that, in the attempt to recall a target word, clouds of related information, on the one hand semantic, on the other hand phonological, are activated and aligned with each other. In doing so, double-activated words can shortly gain aggregate activation above that of the correct alternative and may thus be prone to release [4], [48]. A further interesting result was obtained by investigating the clustering of semantic and phonemic relations independently from each other, without consideration of temporal clusters. In according studies, subjects occasionally produced semantic clusters in phonemic tasks and phonemic clusters in semantic tasks [36], [53], [54], [64], [65]. Such mutually occurring, implicit fluctuations of the cluster category could reflect the retrieval of the demanded lexical data changing with the automatic intrusion of seemingly task-irrelevant information of either type. This seems to speak against dichotomous word processing, implying automatic semantic activation versus attention-demanding phonological functions [55], and is rather in line with the idea of interactive lexical activation spreads.

The present data imply elements of both discrete and interactive lexical processing in that an alignment of word information from both the semantic and the phonological level is organized in sequential intervals. Specifically, the idea that the exchange of information is initiated whenever a semantic field has been entered combines the view of ‘vertical’ word processing with a hierarchy of ‘semantic first’ over secondary phonological operations with a ‘horizontal’ view of parallel processing streams constantly influencing each other.

### Perspectives

In the current study we analysed temporal clusters with respect to phonemic and semantic word relatedness in a letter VF task. The results were interpreted in the framework of influential models of lexical processing. Particularly the view of semantic primacy in lexical processing should be confirmed in future studies by the investigation of phonemic relatedness in semantic VF tasks, complementing the current approach. In so doing, uncertainties from limitations of the present study could at the same time be removed, e. g., by formally controlling potential factors of VF performance, such as intelligence and the gender distribution of study cohorts [66], and by investigating larger study cohorts.

### Conclusion

This first temporal cluster analysis of letter VF supports the idea of a content-based primacy in lexical processing, based on the demonstration that lexico-semantic information invades word production despite the absence of a respective task demand. Besides, the identified dynamics of word relatedness suggest automatic and interactive spread of semantic and phonological word information, as a prerequisite for swiftly performing the given alliteration task. In a nutshell, it is presumed that semantic scanning is the default operation mode during word search for letter VF, and that the exchange with phonological lexical information is restarted whenever a semantic field has been opened.

## Supporting Information

S1 File **Participants.**
(DOCX)

S2 File **Rating Participant.**
(DOCX)

S3 File **Word Relatedness Scores.**
(PDF)
