# Supplementary material for: The Absoluteness of Semantic Processing: Lessons from the Analysis of Temporal Clusters in Phonemic Verbal Fluency
Source: PLoS One. 2014 Dec 23;9(12):e115846. doi: 10.1371/journal.pone.0115846 (PMC4275266; doi:10.1371/journal.pone.0115846)
Supplement: S1 File — Participants. (DOCX) [file pone.0115846.s001.docx]

**Participants S1**

| **Par** | **Age** | **Edu** | **m/f** | **N words** | **Length** | **Interv words** | **Interv intra Cl** | **Interv Inter Cl** | **Slope** | **σ** | **N cl** | **Cl Size** | **N switch** | **Intra cl sem rel** | **Inter cl sem rel** | **Intra cl phon rel** | **Inter cl phon rel** | **Overall sem rel** | **Overall phon rel** |
| --- | --- | --- | --- | --- | --- | --- | --- | --- | --- | --- | --- | --- | --- | --- | --- | --- | --- | --- | --- |
| VF1 | 57 | 13 | m | 34 | 0,81 | 2,69 | 1,31 | 6,73 | 15,40 | 0,87 | 8 | 3,00 | 10 | 1,23 | 0,56 | 0,33 | 0,33 | 1,05 | 0,33 |
| VF2 | 71 | 13 | f | 18 | 0,85 | 5,55 | 1,56 | 10,25 | 18,94 | 0,85 | 3 | 3,33 | 8 | 1,24 | 0,49 | 0,00 | 0,29 | 0,93 | 0,12 |
| VF3 | 63 | 10 | m | 24 | 0,82 | 4,12 | 2,30 | 7,74 | 18,48 | 0,69 | 6 | 2,33 | 10 | 1,08 | 0,09 | 0,14 | 0,33 | 0,69 | 0,22 |
| VF4 | 73 | 13 | m | 18 | 0,67 | 5,76 | 2,51 | 6,78 | 13,30 | 0,29 | 7 | 1,14 | 10 | 0,57 | 0,21 | 0,88 | 0,44 | 0,38 | 0,65 |
| VF5 | 57 | 10 | f | 20 | 0,59 | 5,19 | 3,74 | 10,54 | 11,79 | 1,08 | 2 | 7,00 | 6 | 0,52 | 0,19 | 0,29 | 0,00 | 0,43 | 0,21 |
| VF6 | 55 | 10 | f | 30 | 0,80 | 3,22 | 1,47 | 5,90 | 26,20 | 0,67 | 8 | 2,25 | 12 | 0,72 | 0,12 | 0,39 | 0,00 | 0,49 | 0,24 |
| VF7 | 57 | 10 | m | 28 | 1,14 | 3,10 | 2,40 | 4,42 | 17,90 | 0,42 | 5 | 3,40 | 11 | 0,22 | 0,14 | 0,41 | 0,20 | 0,19 | 0,33 |
| VF8 | 69 | 10 | f | 26 | 0,85 | 3,59 | 1,92 | 6,46 | 12,74 | 0,53 | 8 | 1,88 | 11 | 1,15 | 0,84 | 0,80 | 0,50 | 1,03 | 0,68 |
| VF9 | 77 | 12 | m | 18 | 0,87 | 5,52 | 3,06 | 11,37 | 26,91 | 0,42 | 7 | 1,57 | 7 | 1,01 | 0,21 | 0,55 | 0,17 | 0,73 | 0,41 |
| VF10 | 52 | 9 | m | 34 | 1,01 | 2,53 | 1,77 | 4,26 | 17,45 | 0,53 | 8 | 2,63 | 13 | 0,59 | 0,21 | 0,24 | 0,33 | 0,45 | 0,27 |
| VF11 | 71 | 8 | m | 22 | 0,69 | 4,64 | 3,12 | 7,48 | 10,56 | 0,62 | 7 | 1,86 | 9 | 1,02 | 0,38 | 0,15 | 0,00 | 0,78 | 0,1 |
| VF12 | 71 | 13 | m | 21 | 0,74 | 4,83 | 2,20 | 7,96 | 13,50 | 0,55 | 5 | 2,00 | 11 | 0,20 | 0,36 | 0,20 | 0,00 | 0,28 | 0,1 |
| VF13 | 65 | 13 | f | 53 | 1,16 | 1,11 | 0,69 | 2,21 | 43,66 | 0,74 | 9 | 4,11 | 16 | 0,71 | 0,37 | 0,57 | 0,20 | 0,61 | 0,46 |
| VF14 | 65 | 13 | f | 26 | 0,95 | 3,46 | 2,50 | 5,02 | 23,37 | 0,72 | 6 | 2,67 | 10 | 0,60 | 0,22 | 0,13 | 0,11 | 0,46 | 0,12 |
| VF15 | 71 | 9 | m | 14 | 0,79 | 7,49 | 5,64 | 9,55 | 14,16 | 0,27 | 5 | 1,60 | 6 | 0,75 | 0,64 | 0,13 | 0,00 | 0,7 | 0,08 |
| VF16 | 66 | 13 | m | 45 | 0,77 | 1,90 | 1,41 | 2,95 | 29,81 | 0,47 | 11 | 2,64 | 16 | 0,43 | 0,37 | 0,34 | 0,13 | 0,41 | 0,27 |
| VF17 | 65 | 10 | f | 44 | 1,10 | 1,62 | 0,71 | 2,87 | 27,70 | 0,73 | 9 | 2,67 | 20 | 0,73 | 0,29 | 0,63 | 0,42 | 0,54 | 0,53 |
| VF18 | 66 | 9 | m | 25 | 0,89 | 3,84 | 2,56 | 6,07 | 17,85 | 0,32 | 9 | 1,56 | 11 | 0,47 | 0,13 | 0,71 | 0,50 | 0,33 | 0,63 |
| VF19 | 84 | 13 | m | 36 | 0,75 | 2,55 | 1,31 | 4,09 | 21,62 | 0,50 | 11 | 1,64 | 18 | 0,84 | 0,19 | 0,44 | 0,24 | 0,52 | 0,34 |
| VF20 | 69 | 10 | m | 13 | 0,51 | 8,14 | 4,65 | 12,12 | 17,58 | 0,61 | 4 | 2,25 | 4 | 0,91 | 0,04 | 0,22 | 0,00 | 0,69 | 0,17 |
| VF21 | 77 | 8 | m | 25 | 0,78 | 3,94 | 3,07 | 6,93 | 14,60 | 0,52 | 6 | 2,83 | 8 | 0,80 | 0,71 | 0,53 | 0,29 | 0,78 | 0,46 |
| VF22 | 77 | 8 | m | 16 | 0,83 | 6,32 | 3,35 | 28,22 | 7,74 | 0,92 | 3 | 4,33 | 3 | 1,03 | 1,39 | 0,31 | 0,50 | 1,07 | 0,33 |
| VF23 | 30 | 13 | m | 40 | 0,57 | 2,41 | 1,43 | 3,54 | 27,32 | 0,48 | 10 | 2,30 | 17 | 0,85 | 0,94 | 0,30 | 0,25 | 0,89 | 0,28 |
| VF24 | 34 | 13 | m | 20 | 0,79 | 5,00 | 1,72 | 11,77 | 15,84 | 0,99 | 4 | 3,50 | 6 | 0,52 | 0,49 | 0,07 | 0,40 | 0,51 | 0,16 |
| VF25 | 24 | 13 | f | 31 | 0,66 | 3,16 | 2,53 | 4,71 | 27,35 | 0,69 | 8 | 2,38 | 12 | 0,85 | 0,23 | 0,26 | 0,09 | 0,62 | 0,2 |
| VF26 | 32 | 12 | f | 15 | 0,89 | 6,74 | 4,89 | 13,28 | 12,66 | 0,47 | 4 | 2,75 | 4 | 0,87 | 1,17 | 0,00 | 0,00 | 0,94 | 0 |
| VF27 | 33 | 13 | m | 30 | 1,02 | 2,90 | 1,57 | 4,26 | 21,61 | 0,45 | 7 | 2,29 | 14 | 0,30 | 0,22 | 0,44 | 0,15 | 0,26 | 0,31 |
| VF28 | 30 | 12 | m | 22 | 0,84 | 4,48 | 2,35 | 8,94 | 8,92 | 1,10 | 4 | 3,25 | 9 | 0,64 | 0,27 | 0,23 | 0,00 | 0,5 | 0,14 |
| VF29 | 27 | 13 | f | 22 | 0,54 | 4,74 | 1,99 | 8,35 | 37,83 | 0,67 | 8 | 1,38 | 11 | 0,76 | 0,64 | 0,09 | 0,10 | 0,7 | 0,1 |
| VF30 | 30 | 13 | m | 24 | 0,66 | 4,19 | 2,50 | 8,42 | 11,88 | 0,66 | 5 | 3,40 | 7 | 0,70 | 0,20 | 0,59 | 0,00 | 0,57 | 0,43 |
| VF31 | 38 | 13 | f | 44 | 1,02 | 1,71 | 0,89 | 3,27 | 28,24 | 0,76 | 10 | 2,70 | 17 | 0,46 | 0,26 | 0,15 | 0,19 | 0,39 | 0,16 |
| VF32 | 23 | 12 | f | 40 | 0,78 | 2,21 | 1,18 | 4,21 | 37,68 | 0,59 | 11 | 2,27 | 15 | 0,20 | 0,21 | 0,60 | 0,29 | 0,2 | 0,49 |
| VF33 | 30 | 13 | f | 23 | 0,84 | 4,25 | 2,77 | 5,79 | 34,39 | 0,56 | 8 | 1,50 | 11 | 0,59 | 0,24 | 0,00 | 0,40 | 0,43 | 0,18 |
| VF34 | 46 | 12 | m | 37 | 0,86 | 2,37 | 1,01 | 4,47 | 19,01 | 0,97 | 8 | 2,63 | 16 | 0,43 | 0,35 | 0,48 | 0,20 | 0,4 | 0,36 |
| VF35 | 37 | 13 | f | 25 | 1,08 | 3,63 | 2,08 | 5,74 | 12,28 | 0,82 | 3 | 5,00 | 10 | 0,63 | 0,17 | 0,07 | 0,11 | 0,46 | 0,08 |
| VF36 | 26 | 12 | f | 24 | 0,71 | 4,16 | 2,75 | 8,00 | 19,91 | 0,96 | 5 | 3,40 | 7 | 0,38 | 0,35 | 0,00 | 0,00 | 0,38 | 0 |
| VF37 | 25 | 12 | m | 34 | 0,80 | 2,71 | 1,27 | 5,28 | 27,96 | 0,74 | 7 | 3,29 | 11 | 0,34 | 0,24 | 0,39 | 0,30 | 0,31 | 0,36 |
| VF38 | 32 | 13 | m | 21 | 0,73 | 4,79 | 3,62 | 8,60 | 13,89 | 0,57 | 6 | 2,33 | 7 | 0,54 | 0,46 | 0,21 | 0,00 | 0,52 | 0,15 |
| VF39 | 29 | 13 | m | 24 | 0,85 | 4,16 | 2,67 | 8,18 | 24,60 | 0,60 | 6 | 2,67 | 8 | 0,24 | 0,24 | 0,50 | 0,00 | 0,24 | 0,35 |
| VF40 | 25 | 13 | f | 22 | 0,81 | 4,51 | 1,61 | 11,01 | 26,55 | 0,66 | 5 | 3,00 | 7 | 0,67 | 0,18 | 0,20 | 0,00 | 0,53 | 0,14 |
| VF41 | 35 | 13 | f | 28 | 0,75 | 3,48 | 2,14 | 6,89 | 18,32 | 0,61 | 6 | 3,17 | 9 | 0,33 | 0,23 | 0,47 | 0,25 | 0,3 | 0,41 |
| VF42 | 29 | 13 | f | 39 | 0,52 | 2,53 | 1,76 | 4,00 | 25,75 | 0,64 | 11 | 2,18 | 15 | 0,23 | 0,21 | 0,50 | 0,43 | 0,22 | 0,47 |

*Par* = participants

*Age* = age (years)

*Edu* = level of school education (years)

*m/f* = gender (f = female

m = male)

*N words* = number of words

*Length* = mean word length

*Interv words* = mean word intervals

*Interv intra Cl* = mean intra-cluster word intervals

*Interv Inter Cl* = mean inter-cluster word intervals

*Slope* = slope of exponential curve at t(o)

*σ* = standard deviation of exponential curve-fit

*N cl* = number of clusters

*Cl Size* = mean cluster size

*N switch* = number of switches

*Intra cl sem rel* = intra-cluster semantic word relatedness

*Inter cl sem rel* = inter-cluster semantic word relatedness

*Intra cl phon rel* = intra-cluster phonemic word relatedness

*Inter cl phon rel* = inter-cluster phonemic word relatedness

*Overall sem rel* = overall (inter + intra cluster) semantic word relatedness

*Overall phon rel* = overall (inter + intra cluster) phonemic word relatednes
