# Supplementary material for: The Absoluteness of Semantic Processing: Lessons from the Analysis of Temporal Clusters in Phonemic Verbal Fluency
Source: PLoS One. 2014 Dec 23;9(12):e115846. doi: 10.1371/journal.pone.0115846 (PMC4275266; doi:10.1371/journal.pone.0115846)
Supplement: S2 File — Rating Participant. (DOCX) [file pone.0115846.s002.docx]

**Rating Participants S1**

| **Par** | **Age** | **Edu** | **m/f** | **Sem rating score** | **SD rating score** |
| --- | --- | --- | --- | --- | --- |
| r1 | 33 | 13 | f | 1,7 | 1,39 |
| r2 | 26 | 13 | f | 1 | 1,36 |
| r3 | 33 | 13 | m | 0,88 | 1,42 |
| r4 | 44 | 10 | f | 0,32 | 0,88 |
| r5 | 50 | 12 | f | 0,55 | 1,09 |
| r6 | 50 | 12 | m | 0,89 | 1,37 |
| r7 | 30 | 13 | f | 0,66 | 1,34 |
| r8 | 26 | 13 | f | 0,18 | 0,68 |
| r9 | 30 | 13 | f | 0,39 | 0,91 |
| r10 | 25 | 10 | f | 0,26 | 0,81 |
| r11 | 32 | 11 | m | 0,62 | 1,25 |
| r12 | 67 | 13 | m | 0,7 | 1,28 |
| r13 | 75 | 8 | f | 0,62 | 1,14 |
| r14 | 75 | 8 | m | 0,37 | 0,95 |
| r15 | 40 | 12 | f | 0,4 | 1,02 |
| r16 | 40 | 13 | f | 0,75 | 1,1 |
| r17 | 28 | 13 | f | 0,3 | 0,83 |
| r18 | 68 | 13 | m | 0,24 | 0,69 |
| r19 | 60 | 13 | f | 0,41 | 0,91 |
| r20 | 68 | 12 | f | 0,73 | 1,36 |
| r21 | 72 | 13 | m | 1,15 | 1,44 |
| r22 | 26 | 13 | f | 1,34 | 1,43 |
| r23 | 64 | 12 | m | 0,16 | 0,68 |
| r24 | 68 | 13 | m | 0,51 | 1,08 |
| r25 | 59 | 10 | f | 0,66 | 1,28 |
| r26 | 54 | 13 | f | 0,26 | 0,92 |
| r27 | 60 | 13 | f | 0,32 | 0,82 |
| r28 | 67 | 13 | f | 0,63 | 1,14 |
| r29 | 69 | 13 | f | 0,8 | 1,39 |
| r30 | 70 | 13 | m | 0,89 | 1,34 |

*Par* = participants

*Age* = Age (years)

*Edu* = level of school education (years)

*m/f* = gender (1 = female; 2 = male)

*Sem rating score* = mean rating score of semantic word relatedness

*SD rating score* = standard deviation of the mean rating score of semantic word relatedness
