# Supplementary material for: The Absoluteness of Semantic Processing: Lessons from the Analysis of Temporal Clusters in Phonemic Verbal Fluency
Source: PLoS One. 2014 Dec 23;9(12):e115846. doi: 10.1371/journal.pone.0115846 (PMC4275266; doi:10.1371/journal.pone.0115846)
Supplement: S3 File — Word Relatedness Scores. (PDF) [file pone.0115846.s003.pdf]

Word Relatedness Scores S1

participant	rating of semantic relatedness mean	SD	phonemic relatedness	consecutive words
VF1				
	0,185	0,557	0	Sonne -> Schwein
	0,185	0,786	0	Schwein -> Sozialversicherung
	0,037	0,192	0	Sozialversicherung -> Speisekarte
	0,074	0,267	1	Speisekarte -> Spaßvogel
	0,074	0,267	0	Spaßvogel -> Saftpresse
	0,037	0,192	0	Saftpresse -> Suizid
	0,630	0,884	0	Suizid -> Seelenmassage
	0,704	1,031	0	Seelenmassage -> Seifenoper
	0,074	0,385	1	Seifenoper -> Seil
	0,037	0,192	0	Seil -> Sender
	0,000	0,000	1	Sender -> Sellerie
	0,037	0,192	0	Sellerie -> Sommerreifen
	0,185	0,681	0	Sommerreifen -> Salat
	2,667	1,209	0	Salat -> Spinat
	0,444	0,847	0	Spinat -> Satsuma
	0,000	0,000	0	Satsuma -> Sitz
	3,667	0,620	0	Sitz -> Stuhl
	3,333	0,920	0	Stuhl -> Sofa
	3,556	0,801	0	Sofa -> Sessel
	1,000	1,074	0	Sessel -> Seide
	3,444	0,847	1	Seide -> Samt
	0,259	0,526	0	Samt -> Softdrink
	0,556	0,974	0	Softdrink -> schnell
	3,556	0,577	0	schnell -> spurten
	3,741	0,526	1	spurten -> Sprint
	0,370	0,926	1	Sprint -> Spezialabteilung
	0,370	0,742	1	Spezialabteilung -> Spender
	2,778	1,188	1	Spender -> Sponsor
	0,333	0,734	1	Sponsor -> Sparring
	0,037	0,192	0	Sparring -> schreiben
	0,296	0,669	0	schreiben -> stehlen
	0,074	0,267	1	stehlen -> stehen
	1,926	1,357	1	stehen -> Stufe
VF2				
	0,778	1,121	0	Schokolade -> Salbei
	1,037	1,192	0	Salbei -> Süßigkeiten
	2,370	1,182	0	Süßigkeiten -> Sahne
	1,593	1,366	0	Sahne -> Sirup
	0,692	1,158	0	Sirup -> Sojamehl
	1,148	1,322	0	Sojamehl -> Sauerteig
	0,667	1,240	0	Sauerteig -> Schnitzel
	1,741	1,457	0	Schnitzel -> Senf

	0,148	0,456	1 Senf -> Sägemehl
	0,185	0,557	0 Sägemehl -> Steckrübe
	0,815	1,075	0 Steckrübe -> Süßigkeiten
	0,111	0,424	0 Süßigkeiten -> Sänger
	0,370	0,742	1 Sänger -> Sessel
	3,296	0,953	0 Sessel -> Sofa
	0,148	0,456	0 Sofa -> Sägemehl
	0,704	0,953	0 Sägemehl -> Sand
	0,037	0,192	0 Sand -> Suppe
VF3			
	0,296	0,609	0 Straße -> Sau
	3,778	0,641	0 Sau -> Schwein
	0,037	0,192	0 Schwein -> Stufe
	1,222	1,155	0 Stufe -> Sitz
	0,111	0,320	0 Sitz -> Sterne
	0,000	0,000	1 Sterne -> Stamm
	0,296	0,669	1 Stamm -> Straßenbahn
	3,259	0,903	0 Straßenbahn -> S-Bahn
	0,963	1,581	0 S-Bahn -> Südstern
	0,111	0,577	0 Südstern -> Stulle
	0,074	0,385	1 Stulle -> Sturheit
	0,556	1,013	0 Sturheit -> Seriosität
	0,037	0,192	0 Seriosität -> Stecken
	2,704	1,750	1 Stecken -> Stab
	0,222	0,577	0 Stab -> Singvogel
	1,111	1,121	0 Singvogel -> Säbelzahntiger
	0,000	0,000	0 Säbelzahntiger -> S-Phon (=Eigenname Handy)
	0,000	0,000	0 S-Phon (=Eigenname Handy) -> Stanniolpapier
	0,074	0,267	1 Stanniolpapier -> Strang
	0,037	0,192	0 Strang -> Sabber
	0,519	0,935	0 Sabber -> Süßstoff
	0,000	0,000	0 Süßstoff -> Sauzahn
	0,556	1,219	0 Sauzahn -> Spaten
VF4			
	0,148	0,602	1 Sau -> Saft
	0,556	0,892	1 Saft -> Samen
	0,148	0,602	1 Samen -> Sage
	0,185	0,622	0 Sage -> Segel
	2,222	1,553	1 Segel -> See
	0,333	0,784	0 See -> Sinn
	0,593	0,888	1 Sinn -> Sicht
	0,074	0,385	1 Sicht -> sieben
	0,593	1,248	1 sieben -> Sinn
	0,148	0,602	0 Sinn -> Sofa
	0,407	0,797	1 Sofa -> Sonne
	0,185	0,557	0 Sonne -> Samurai
	0,185	0,622	1 Samurai -> Salon
	0,037	0,192	0 Salon -> Sud

	0,333	0,832	0 Sud -> Suff
	0,148	0,456	1 Suff -> Summe
	0,148	0,770	1 Summe -> Sumpf
VF5			
	0,444	0,751	0 Schiff -> Solaranlage
	0,000	0,000	0 Solaranlage -> Semmel
	1,148	1,199	0 Semmel -> Sauce
	2,222	1,601	0 Sauce -> Suppe
	0,037	0,192	0 Suppe -> Serenade
	0,000	0,000	0 Serenade -> Sahne
	0,000	0,000	0 Sahne -> Surfboard
	0,259	0,656	0 Surfboard -> Sänger
	0,148	0,456	0 Sänger -> Sülze
	0,481	0,802	0 Sülze -> Schimmel
	0,037	0,192	0 Schimmel -> Sender
	0,037	0,192	0 Sender -> Seil
	0,000	0,000	0 Seil -> Selen
	0,000	0,000	0 Selen -> Ski
	1,074	1,439	0 Ski -> Schal
	0,037	0,192	2 Schal -> Schall
	1,000	1,359	0 Schall -> Schlager
	0,222	0,641	1 Schlager -> Schluck
	1,038	1,148	1 Schluck -> Schleim
VF6			
	0,815	1,039	0 Sonne -> singen
	0,593	0,888	0 singen -> Sarg
	0,000	0,000	0 Sarg -> Steppe
	0,037	0,192	0 Steppe -> Sirene
	0,259	0,712	0 Sirene -> Saal
	0,370	0,839	0 Saal -> summen
	0,296	0,609	0 summen -> schlucken
	0,000	0,000	0 schlucken -> schießen
	0,000	0,000	0 schießen -> schlafen
	0,111	0,320	1 schlafen -> schlendern
	0,074	0,385	0 schlendern -> Schrank
	0,000	0,000	0 Schrank -> Schelm
	0,037	0,192	0 Schelm -> schwimmen
	0,481	0,893	1 schwimmen -> Schwanz
	0,000	0,000	0 Schwanz -> Sieger
	0,037	0,192	0 Sieger -> Schlumpf
	0,000	0,000	1 Schlumpf -> Schleppe
	0,259	0,594	1 Schleppe -> schleichen
	0,074	0,267	0 schleichen -> sagen
	2,074	1,385	1 sagen -> Sage
	0,259	0,594	0 Sage -> sichern
	2,926	1,328	0 sichern -> Schloss
	3,630	0,742	1 Schloss -> schließen
	0,296	0,953	0 schließen -> schön

0,778	1,013	0	schön -> schräg
0,185	0,483	0	schräg -> schlafen
0,407	0,694	1	schlafen -> schlucken
0,000	0,000	0	schlucken -> Schein
0,333	0,734	0	Schein -> Schule

VF7

0,222	0,847	0	Sause -> Stafette
0,148	0,456	0	Stafette -> Sabine
0,037	0,192	0	Sabine -> Sache
1,074	1,107	0	Sache -> Sorte
0,037	0,192	0	Sorte -> Sakrileg
0,074	0,385	0	Sakrileg -> Sortiment
0,333	0,734	1	Sortiment -> Sauce
0,407	0,888	0	Sauce -> Salbe
0,074	0,385	1	Salbe -> Savanne
0,259	0,656	0	Savanne -> Sicherheit
0,296	0,669	0	Sicherheit -> Subunternehmen
0,000	0,000	1	Subunternehmen -> Sulky
0,000	0,000	1	Sulky -> Summa summarum
0,000	0,000	0	Summa summarum -> Saft
0,333	0,920	1	Saft -> Sause
0,037	0,192	0	Sause -> Staubsauger
0,000	0,000	0	Staubsauger -> Silizium
1,000	1,468	1	Silizium -> Silikon
0,037	0,192	0	Silikon -> Säbel
0,000	0,000	0	Säbel -> Sauce
0,000	0,000	0	Sauce -> Sakrileg
0,444	0,801	1	Sakrileg -> Sarkophag
0,074	0,385	1	Sarkophag -> Salmonellen
0,222	0,506	0	Salmonellen -> Sense
0,037	0,192	0	Sense -> Seismograph
0,000	0,000	1	Seismograph -> Seife
0,038	0,196	0	Seife -> Solei

VF8

3,370	0,792	1	Sonne -> Sommer
0,111	0,577	0	Sommer -> Silbe
0,074	0,385	0	Silbe -> selbige
3,667	1,074	1	selbige -> selbige
0,148	0,456	0	selbige -> stumm
0,667	1,074	1	stumm -> steif
0,630	1,115	1	steif -> Stille
0,778	1,013	1	Stille -> Strand
1,741	1,607	0	Strand -> schön
0,259	0,813	2	schön -> schon
0,000	0,000	0	schon -> schummeln
0,000	0,000	2	schummeln -> schimmeln
0,074	0,267	0	schimmeln -> Schemel
0,074	0,267	0	Schemel -> Scham

	3,667	0,832	0 Scham -> schämen
	0,074	0,385	0 schämen -> Sage
	2,185	1,360	1 Sage -> sagen
	0,185	0,483	1 sagen -> sammeln
	0,259	0,594	1 sammeln -> sausen
	0,111	0,577	1 sausen -> Sau
	3,815	0,622	0 Sau -> Schwein
	1,481	1,341	1 Schwein -> Schwalbe
	0,074	0,267	1 Schwalbe -> schwitzen
	1,852	1,379	0 schwitzen -> schmoren
	0,370	0,792	2 schmoren -> schmieren
VF9			
	0,481	0,849	0 Schuster -> Schiffsarzt
	0,185	0,483	0 Schiffsarzt -> Schule
	3,630	0,629	1 Schule -> Schulhaus
	0,370	0,688	0 Schulhaus -> schwimmen
	0,111	0,424	0 schwimmen -> Spaten
	0,000	0,000	1 Spaten -> sprechen
	1,111	1,086	1 sprechen -> spielen
	1,148	1,292	1 spielen -> Sprache
	0,148	0,602	0 Sprache -> Schutzmantel
	0,481	1,087	0 Schutzmantel -> Schütze
	0,074	0,267	1 Schütze -> Schirmer
	0,000	0,000	0 Schirmer -> schnell
	0,296	0,775	0 schnell -> schlecht
	1,370	1,621	0 schlecht -> Schimmel
	0,231	0,587	0 Schimmel -> schlafen
	1,704	1,382	1 schlafen -> schlapp
	1,000	1,209	1 schlapp -> schlimm
VF10			
	0,407	0,797	0 Suizid -> sorglos
	0,148	0,456	0 sorglos -> Servus
	2,667	1,441	0 Servus -> Salute
	0,370	0,792	0 Salute -> Solidarität
	0,148	0,602	0 Solidarität -> Sakrament
	0,111	0,424	0 Sakrament -> Sozius
	0,111	0,577	0 Sozius -> Schwein
	0,074	0,267	0 Schwein -> sonstwas
	0,037	0,192	0 sonstwas -> Sahne
	0,259	0,764	0 Sahne -> sorglos
	0,037	0,192	0 sorglos -> systematisch
	0,704	1,235	1 systematisch -> sinngemäß
	0,000	0,000	0 sinngemäß -> Serpentine
	0,111	0,320	0 Serpentine -> sieben
	0,037	0,192	0 sieben -> Sabine
	0,074	0,267	0 Sabine -> Segen
	0,111	0,320	0 Segen -> sachte
	0,037	0,192	1 sachte -> Sache

	0,000	0,000	1 Sache -> Sahib (Marokkanischer Name)
	0,037	0,192	1 Sahib (Marokkanischer Name) -> seicht
	1,407	1,217	0 seicht -> See
	0,074	0,385	0 See -> Saal
	1,074	1,174	1 Saal -> Samba
	0,407	0,694	0 Samba -> sentimental
	1,037	1,224	0 sentimental -> Silvester
	0,148	0,362	0 Silvester -> strategisch
	0,111	0,320	1 strategisch -> Stratosphäre
	1,593	1,575	0 Stratosphäre -> Sonne
	2,630	1,334	0 Sonne -> Saturn
	0,148	0,534	0 Saturn -> sterben
	0,185	0,622	1 sterben -> stehen
	0,333	0,620	1 stehen -> Stollen
	0,222	0,641	1 Stollen -> Strafe
VF11			
	0,259	0,656	0 Schiff -> Schloss
	1,889	1,867	0 Schloss -> Schüssel
	0,296	0,724	0 Schüssel -> Stand
	1,370	1,548	1 Stand -> Stau
	0,333	1,074	0 Stau -> Schwein
	0,222	0,506	0 Schwein -> Straße
	0,296	0,542	0 Straße -> Schaufel
	0,630	1,115	0 Schaufel -> Schraube
	0,074	0,267	0 Schraube -> Salat
	0,000	0,000	0 Salat -> Sorge
	1,259	1,289	0 Sorge -> Sicherheit
	0,593	1,118	0 Sicherheit -> Sanssouci
	0,704	1,203	0 Sanssouci -> Soldat
	2,852	1,486	0 Soldat -> Sergeant
	0,185	0,681	0 Sergeant -> Sozietät
	0,963	1,315	1 Sozietät -> Solidarität
	0,148	0,534	0 Solidarität -> Souffleuse
	2,667	1,387	0 Souffleuse -> Schauspieler
	0,296	0,724	0 Schauspieler -> Skat
	1,185	1,415	0 Skat -> Salon
	0,077	0,392	0 Salon -> Sauce
VF12			
	1,000	1,209	0 Säge -> Schwert
	0,074	0,385	0 Schwert -> Socke
	0,074	0,385	0 Socke -> sieben
	0,037	0,192	0 sieben -> seicht
	0,074	0,385	0 seicht -> super
	0,963	1,427	0 super -> schwer
	0,741	1,289	0 schwer -> super
	0,815	1,388	0 super -> sechs
	0,000	0,000	0 sechs -> sauber
	0,185	0,622	0 sauber -> Sieg

0,148	0,362	0 Sieg -> Schwiele
0,185	0,622	0 Schwiele -> Schurke
0,074	0,267	0 Schurke -> sedierend
0,148	0,534	0 sedierend -> Sud
0,000	0,000	2 Sud -> süd
0,222	0,698	0 süd -> saufen
0,481	1,014	0 saufen -> sehr
0,185	0,622	0 sehr -> Schuld
0,000	0,000	0 Schuld -> sieben (Verb)
0,148	0,456	0 sieben (Verb) -> schweben

VF13

0,630	0,926	1 Sturm -> Stiefel
0,111	0,577	1 Stiefel -> Stamm
0,407	0,971	1 Stamm -> Stolz
0,148	0,534	0 Stolz -> Schienbein
0,111	0,424	0 Schienbein -> Schlange
0,111	0,320	0 Schlange -> Schubkarre
0,444	0,847	0 Schubkarre -> Scheiße
0,074	0,267	0 Scheiße -> Schlinge
0,963	1,480	0 Schlinge -> Sturz
3,000	0,832	1 Sturz -> stolpern
0,148	0,456	0 stolpern -> sagen
3,407	1,047	0 sagen -> sprechen
2,000	1,209	0 sprechen -> säuseln
1,444	1,281	0 säuseln -> singen
0,074	0,385	0 singen -> segeln
0,037	0,192	0 segeln -> stehlen
3,481	1,122	1 stehlen -> stibitzen
0,111	0,320	1 stibitzen -> sträuben
0,074	0,385	1 sträuben -> Strauch
0,259	0,712	1 Strauch -> Strieme
0,185	0,786	1 Strieme -> streben
0,185	0,557	1 streben -> Sturz
0,074	0,385	0 Sturz -> Samen
3,481	0,935	1 Samen -> Saat
0,111	0,424	0 Saat -> Sud
1,074	1,412	0 Sud -> Sieb
0,074	0,385	0 Sieb -> Segen
1,556	1,553	1 Segen -> Seele
0,630	1,043	1 Seele -> Seemann
0,444	0,751	0 Seemann -> summen
1,370	1,214	0 summen -> sprechen
0,037	0,192	1 sprechen -> spiegeln
0,259	0,859	1 spiegeln -> spielen
0,148	0,456	1 spielen -> Spaten
0,111	0,424	1 Spaten -> spät
0,074	0,385	1 spät -> Sporen
0,926	1,299	0 Sporen -> Säbel
0,185	0,483	0 Säbel -> suchen

0,185	0,622	1 suchen -> Substantiv
0,037	0,192	0 Substantiv -> so
0,111	0,424	1 so -> Sole
0,074	0,385	0 Sole -> Sport
1,111	1,423	1 Sport -> Spitze
1,259	1,430	1 Spitze -> Spritze
0,185	0,681	1 Spritze -> spannend
0,222	0,698	0 spannend -> Schub
0,185	0,786	0 Schub -> Schere
0,000	0,000	0 Schere -> schade
0,000	0,000	0 schade -> Schober
0,037	0,192	1 Schober -> schonen
0,222	0,577	0 schonen -> Sonne
0,115	0,431	0 Sonne -> sieben

VF14

2,259	1,509	0 See -> Sand
0,519	1,122	0 Sand -> Stern
2,815	1,415	0 Stern -> Sonne
0,148	0,534	0 Sonne -> sammeln
0,148	0,456	0 sammeln -> sickern
0,222	0,577	0 sickern -> suchen
0,296	0,669	0 suchen -> Salat
0,000	0,000	0 Salat -> Sirene
0,889	1,340	0 Sirene -> sausen
0,037	0,192	0 sausen -> sortieren
0,481	0,802	0 sortieren -> servieren
0,185	0,622	0 servieren -> suchen
0,963	1,344	1 suchen -> Sucht
0,000	0,000	0 Sucht -> Sims
0,148	0,456	0 Sims -> Sonaten
0,037	0,192	0 Sonaten -> selektieren
0,000	0,000	0 selektieren -> summen
0,000	0,000	0 summen -> siechen
0,000	0,000	0 siechen -> schreiben
0,259	0,594	0 schreiben -> Stärke
0,148	0,362	0 Stärke -> Schlamm
1,407	1,448	1 Schlamm -> Schlieren
0,000	0,000	1 Schlieren -> Schlaraffenland
0,556	1,050	0 Schlaraffenland -> Sofa
0,037	0,192	0 Sofa -> Sarkasmus

VF15

0,556	1,013	0 Stadt -> Sensation
1,481	1,312	0 Sensation -> super
1,333	1,687	0 super -> sechs
0,296	0,724	0 sechs -> Systematik
0,704	0,953	0 Systematik -> Summe
0,926	1,207	0 Summe -> Schule
1,222	1,311	0 Schule -> Stadt

0,593	1,010	0	Stadt -> Sensation
1,444	1,450	0	Sensation -> super
0,481	1,051	0	super -> Schnellstraße
0,037	0,192	0	Schnellstraße -> Stuhl
0,000	0,000	0	Stuhl -> Samen
0,074	0,267	1	Samen -> Seife

VF16

0,630	0,884	0	Stau -> schwierig
2,889	1,251	1	schwierig -> schwer
0,407	0,844	0	schwer -> stammeln
0,000	0,000	1	stammeln -> Steuer
0,148	0,602	1	Steuer -> Stigma
0,333	0,961	1	Stigma -> starr
0,111	0,424	0	starr -> selbst
1,667	1,732	0	selbst -> süchtig
0,111	0,320	0	süchtig -> staunen
0,037	0,192	0	staunen -> schwimmen
0,296	0,669	0	schwimmen -> schreien
1,444	1,396	0	schreien -> spielen
0,852	1,167	0	spielen -> schlagen
0,037	0,192	0	schlagen -> schwärmen
0,000	0,000	0	schwärmen -> stanzen
0,000	0,000	1	stanzen -> stolpern
0,481	0,849	1	stolpern -> stutzen
0,000	0,000	1	stutzen -> stinken
0,000	0,000	0	stinken -> schlagen
1,148	1,199	1	schlagen -> schleudern
0,074	0,267	0	schleudern -> schwimmen
1,074	1,328	0	schwimmen -> sauber
0,741	1,095	0	sauber -> sehr
0,704	1,203	0	sehr -> sagenhaft
0,741	1,196	0	sagenhaft -> schrecklich
0,593	0,931	0	schrecklich -> schlau
0,407	0,888	0	schlau -> stur
0,000	0,000	0	stur -> Schanze
0,000	0,000	0	Schanze -> Schimmel
0,407	0,888	0	Schimmel -> Schweiß
0,222	0,801	0	Schweiß -> Schlag
0,000	0,000	0	Schlag -> spinnen
0,148	0,456	0	spinnen -> strecken
0,185	0,483	1	strecken -> staunen
0,259	0,656	0	staunen -> Schuss
0,148	0,602	0	Schuss -> schwitzen
0,185	0,681	0	schwitzen -> stürzen
0,222	0,506	1	stürzen -> Stab
0,074	0,385	1	Stab -> streuen
0,037	0,192	0	streuen -> Silber
0,000	0,000	0	Silber -> sengen
0,000	0,000	0	sengen -> super

	0,037	0,192	0 super -> Salz
	1,185	1,415	1 Salz -> Sack
VF17			
	0,593	0,888	0 speziell -> Superlativ
	0,000	0,000	1 Superlativ -> Suppe
	0,074	0,385	0 Suppe -> Synonym
	0,074	0,385	0 Synonym -> Sarg
	0,037	0,192	0 Sarg -> sähen
	0,185	0,483	3 sähen -> sägen
	0,333	0,734	0 sägen -> Sport
	2,000	1,177	1 Sport -> spielen
	1,889	1,396	1 spielen -> springen
	0,111	0,424	0 springen -> sabotieren
	0,148	0,456	0 sabotieren -> Stockwerk
	0,111	0,424	1 Stockwerk -> Stamm
	0,259	0,656	1 Stamm -> Straße
	0,259	0,526	1 Straße -> Studio
	0,111	0,577	1 Studio -> stagnieren
	0,593	1,083	1 stagnieren -> stottern
	1,222	1,281	1 stottern -> stolpern
	0,148	0,456	1 stolpern -> Strategie
	0,111	0,424	1 Strategie -> sterben
	0,000	0,000	1 sterben -> stanzen
	0,370	0,792	1 stanzen -> stapeln
	0,407	0,844	0 stapeln -> schneiden
	0,519	1,051	0 schneiden -> Schule
	0,259	0,712	0 Schule -> Sonne
	0,222	0,698	0 Sonne -> Startfeld
	0,185	0,681	1 Startfeld -> Stigma
	0,185	0,786	1 Stigma -> stramm
	0,296	0,609	0 stramm -> singen
	1,148	1,350	0 singen -> saufen
	0,333	0,734	0 saufen -> seufzen
	0,370	0,926	0 seufzen -> Silbe
	0,037	0,192	1 Silbe -> sichern
	0,593	0,931	0 sichern -> suchen
	0,185	0,681	1 suchen -> super
	0,185	0,681	0 super -> sorgen
	0,000	0,000	0 sorgen -> spachteln
	1,593	1,526	0 spachteln -> schleifen
	0,852	1,350	0 schleifen -> spalten
	1,185	1,302	0 spalten -> schlagen
	2,593	1,118	0 schlagen -> stoßen
	0,259	0,656	1 stoßen -> stramm
	0,185	0,483	1 stramm -> Stramin (=Stoff zum Sticken)
	2,889	1,625	1 Stramin (=Stoff zum Sticken) -> sticken
VF18			
	0,074	0,385	0 spielen -> Strauß

	0,074	0,267	1 Strauß -> Straße
	0,296	0,823	1 Straße -> Stock
	1,630	1,445	1 Stock -> Stein
	0,296	0,869	1 Stein -> Sturm
	0,074	0,385	0 Sturm -> Suppe
	0,148	0,602	0 Suppe -> Sisyphos
	0,037	0,192	1 Sisyphos -> Sinus
	0,185	0,622	0 Sinus -> Steige
	1,704	1,613	2 Steige -> Stiege
	0,037	0,192	1 Stiege -> Stolz
	0,111	0,320	1 Stolz -> stehlen
	0,148	0,362	1 stehlen -> stoppen
	0,074	0,385	1 stoppen -> Stiel
	0,074	0,267	1 Stiel -> Stoff
	0,370	0,742	0 Stoff -> Säure
	0,111	0,424	0 Säure -> Stab
	0,296	0,724	1 Stab -> stopp
	0,333	0,877	1 stopp -> stoßen
	0,111	0,320	0 stoßen -> senil
	0,037	0,192	0 senil -> Sturm
	1,519	1,477	0 Sturm -> sausen
	0,074	0,267	0 sausen -> Stimme
	0,000	0,000	1 Stimme -> stehlen
VF19			
	0,074	0,267	0 Schule -> Salz
	0,630	0,967	1 Salz -> Sand
	0,222	0,698	0 Sand -> selbständig
	0,704	1,103	0 selbständig -> Studie
	0,667	1,240	0 Studie -> Sport
	0,630	1,079	0 Sport -> Stadt
	0,148	0,456	0 Stadt -> süß
	2,444	1,502	0 süß -> sauer
	0,074	0,385	0 sauer -> Socke
	1,630	1,471	0 Socke -> Schlips
	2,259	1,289	0 Schlips -> Schal
	0,074	0,385	0 Schal -> Salz
	0,037	0,192	0 Salz -> Spott
	0,074	0,385	0 Spott -> Schuh
	0,037	0,192	0 Schuh -> Chan
	0,000	0,000	1 Chan -> Schaden
	0,222	0,801	0 Schaden -> Suppe
	0,074	0,267	0 Suppe -> Satire
	0,296	0,775	0 Satire -> Sofa
	2,852	1,231	0 Sofa -> Stuhl
	0,111	0,424	1 Stuhl -> Stoa
	0,333	0,734	1 Stoa -> Studium
	0,556	1,050	1 Studium -> Standpunkt
	0,148	0,534	1 Standpunkt -> Strolch
	0,148	0,534	1 Strolch -> Stroh

	0,111	0,424	1 Stroh -> Staat
	1,963	1,255	2 Staat -> Stadt
	0,296	0,724	1 Stadt -> stetig
	0,148	0,456	0 stetig -> sauer
	0,259	0,712	0 sauer -> Sumpf
	0,037	0,192	0 Sumpf -> Sonde
	0,000	0,000	0 Sonde -> sämig
	0,926	1,238	0 sämig -> Sahne
	0,000	0,000	0 Sahne -> Spalt
	0,148	0,770	1 Spalt -> Spleen
VF20			
	1,778	1,093	0 Schnee -> Sonne
	0,000	0,000	0 Sonne -> Sache
	0,111	0,333	0 Sache -> süß
	2,667	1,414	0 süß -> sauer
	0,000	0,000	0 sauer -> schlafen
	1,778	1,481	0 schlafen -> schön
	1,000	1,118	0 schön -> selten
	0,111	0,333	0 selten -> Säule
	0,000	0,000	1 Säule -> Socken
	0,111	0,333	0 Socken -> Siphon
	0,333	0,707	1 Siphon -> sicher
	0,444	1,014	0 sicher -> suchen
VF21			
	0,556	0,882	0 Schnee -> Spiegel
	1,333	1,500	0 Spiegel -> Seife
	0,667	0,866	0 Seife -> Solarium
	3,556	0,726	1 Solarium -> Sonne
	0,667	1,000	0 Sonne -> Silizium
	0,000	0,000	0 Silizium -> Segel
	0,000	0,000	0 Segel -> Singular
	0,000	0,000	0 Singular -> Sohn
	2,889	1,167	0 Sohn -> Schwiegermutter
	0,000	0,000	0 Schwiegermutter -> Schein
	0,000	0,000	0 Schein -> Spriegel (=Metallbügel)
	0,111	0,333	0 Spriegel (=Metallbügel) -> Sofa
	3,667	0,500	0 Sofa -> Sessel
	0,000	0,000	0 Sessel -> steil
	1,778	1,394	1 steil -> Stiege
	0,000	0,000	1 Stiege -> Strafe
	0,222	0,441	1 Strafe -> Stellung
	0,000	0,000	1 Stellung -> steril
	0,000	0,000	1 steril -> Strom
	0,333	0,707	1 Strom -> Steigung
	0,778	1,093	1 Steigung -> Stelze
	1,222	1,394	1 Stelze -> Stiefel
	0,667	0,866	1 Stiefel -> Stufe
	0,222	0,667	1 Stufe -> Stall

VF22

1,000	1,118	0	Sieg -> Sage
0,556	1,130	0	Sage -> Sohn
0,000	0,000	0	Sohn -> Sansibar
0,111	0,333	0	Sansibar -> singen
0,778	1,093	3	singen -> springen
2,111	1,167	1	springen -> spielen
2,667	0,866	1	spielen -> Sport
1,778	1,563	0	Sport -> Schach
0,667	0,866	0	Schach -> schwitzen
1,111	1,054	0	schwitzen -> Saft
0,222	0,667	0	Saft -> Schrank
1,889	1,269	0	Schrank -> Stuhl
3,000	0,707	0	Stuhl -> Sofa
0,000	0,000	0	Sofa -> Segen
0,222	0,441	0	Segen -> sicherstellen

VF23

2,481	1,424	0	Schuh -> Schrank
1,444	1,368	0	Schrank -> Strumpf
0,037	0,192	0	Strumpf -> Schleifpapier
0,037	0,192	0	Schleifpapier -> Sonnenbrille
0,444	0,751	0	Sonnenbrille -> Stiefel
0,185	0,622	0	Stiefel -> Sau
2,148	1,379	0	Sau -> Stier
0,185	0,622	0	Stier -> Sauberkeit
2,593	1,421	0	Sauberkeit -> Schmutz
2,148	1,199	0	Schmutz -> Streifen
3,000	1,177	1	Streifen -> Striemen
1,296	1,514	1	Striemen -> Strapse
0,370	0,926	1	Strapse -> stehen
2,222	1,311	1	stehen -> Standbild
1,111	1,251	1	Standbild -> Status
1,370	1,214	0	Status -> Situation
0,185	0,483	1	Situation -> Sirene
0,259	0,656	0	Sirene -> schlau
0,889	1,121	0	schlau -> sicher
1,185	1,272	0	sicher -> super
0,333	0,679	0	super -> sensibel
0,111	0,424	0	sensibel -> Stau
1,444	1,281	0	Stau -> schlimm
0,407	1,010	1	schlimm -> schlau
0,037	0,192	0	schlau -> Sichel
0,815	1,145	1	Sichel -> Silber
0,667	0,920	0	Silber -> Samt
0,148	0,602	0	Samt -> Supermarkt
0,000	0,000	0	Supermarkt -> Silbe
0,037	0,192	0	Silbe -> Sturm
0,111	0,424	0	Sturm -> Salbe

	1,111	1,219	1 Salbe -> Salbei
	0,111	0,424	0 Salbei -> Suche
	0,222	0,577	0 Suche -> schwimmen
	0,074	0,267	0 schwimmen -> Stift
	1,963	1,400	0 Stift -> Schablone
	0,370	0,742	0 Schablone -> Stein
	1,519	1,477	1 Stein -> Stock
	1,556	1,423	1 Stock -> stabil
VF24			
	0,038	0,196	1 Sonne -> Sauce
	0,074	0,267	0 Sauce -> Schein
	1,259	1,403	0 Schein -> Silber
	0,074	0,267	0 Silber -> Sud
	0,630	1,149	0 Sud -> Sahne
	0,556	1,013	0 Sahne -> Säure
	0,074	0,385	0 Säure -> sinken
	1,963	1,400	2 sinken -> senken
	0,519	0,975	0 senken -> schnell
	0,148	0,534	0 schnell -> Scheiße
	0,148	0,534	0 Scheiße -> sind
	0,185	0,557	0 sind -> suchen
	2,111	1,281	0 suchen -> sondieren
	1,296	1,265	0 sondieren -> sammeln
	0,148	0,456	0 sammeln -> Seuche
	0,148	0,456	0 Seuche -> Sinnbild
	0,222	0,641	0 Sinnbild -> Summe
	0,037	0,192	0 Summe -> Samen
	0,037	0,192	0 Samen -> System
VF25			
	0,185	0,622	0 Schwein -> Stadt
	0,630	0,926	0 Stadt -> spielen
	0,296	0,669	0 spielen -> Sau
	0,481	0,893	0 Sau -> Suppe
	2,963	1,160	0 Suppe -> speisen
	1,222	1,502	0 speisen -> super
	1,185	1,520	0 super -> Spaghetti
	0,185	0,396	0 Spaghetti -> Säure
	1,370	1,418	0 Säure -> Sprudel
	0,111	0,320	0 Sprudel -> Salbei
	0,000	0,000	1 Salbei -> sausen
	1,481	1,189	0 sausen -> springen
	1,667	1,330	0 springen -> schwimmen
	0,704	1,325	0 schwimmen -> Spitze
	0,111	0,424	0 Spitze -> surreal
	0,000	0,000	0 surreal -> Speer
	0,074	0,267	0 Speer -> Sieb
	0,296	0,669	0 Sieb -> schwer
	0,222	0,698	0 schwer -> Spritze

0,630	1,149	0 Spritze -> Saboteur
0,000	0,000	1 Saboteur -> seicht
0,074	0,267	0 seicht -> sprechen
3,407	1,010	0 sprechen -> schwatzen
0,148	0,456	0 schwatzen -> sinken
0,148	0,602	0 sinken -> Satzung
0,000	0,000	1 Satzung -> Seide
0,111	0,424	0 Seide -> Staudamm
0,296	0,669	1 Staudamm -> Stück
0,037	0,192	1 Stück -> Star
0,556	1,050	1 Star -> stumm

VF26

0,481	1,014	0 Sonne -> Schule
1,185	1,241	0 Schule -> schwimmen
3,037	1,224	0 schwimmen -> Strand
2,333	1,209	0 Strand -> Sandkasten
0,444	1,013	0 Sandkasten -> Straße
0,963	1,126	0 Straße -> Schatten
1,704	1,564	0 Schatten -> suchen
2,037	1,427	0 suchen -> sortieren
0,037	0,192	0 sortieren -> Sandmännchen
0,000	0,000	0 Sandmännchen -> sondieren
0,037	0,192	0 sondieren -> Stuhl
0,370	0,792	0 Stuhl -> Spüle
0,407	0,797	0 Spüle -> Stehlampe
0,074	0,267	0 Stehlampe -> Sprudelwasser

VF27

0,259	0,712	0 Sonne -> Seide
0,074	0,385	1 Seide -> Sand
2,074	1,615	0 Sand -> Sturm
0,222	0,577	1 Sturm -> Ständer
0,000	0,000	0 Ständer -> Sage
0,037	0,192	0 Sage -> Straße
0,222	0,506	1 Straße -> Stamm
0,259	0,447	1 Stamm -> Strand
0,852	1,134	0 Strand -> Schutz
0,037	0,192	0 Schutz -> schon
0,037	0,192	0 schon -> Sieg
0,037	0,192	0 Sieg -> steif
0,037	0,192	1 steif -> strahlen
0,185	0,483	1 strahlen -> stehen
1,222	1,188	1 stehen -> straucheln
0,333	0,679	0 straucheln -> schauen
0,407	0,888	0 schauen -> senden
0,000	0,000	0 senden -> Sau
0,000	0,000	0 Sau -> Silber
0,259	0,594	0 Silber -> Schund
0,407	0,844	0 Schund -> schön

	0,148	0,456	0 schön -> Standard
	0,000	0,000	1 Standard -> strullern
	0,148	0,362	0 strullern -> Spiel
	0,000	0,000	0 Spiel -> Sülze
	0,000	0,000	0 Sülze -> Summe
	0,037	0,192	0 Summe -> sondern
	0,037	0,192	0 sondern -> Sinn
	0,259	0,594	1 Sinn -> Sünde
VF28			
	1,407	1,394	0 Sieg -> Schütze
	0,000	0,000	0 Schütze -> Saft
	0,074	0,267	0 Saft -> Schiff
	0,074	0,267	0 Schiff -> Salat
	0,000	0,000	0 Salat -> Schreiber
	0,037	0,192	1 Schreiber -> schreien
	0,481	1,014	0 schreien -> S-Bahn
	0,222	0,577	0 S-Bahn -> Sport
	0,037	0,192	0 Sport -> Siegelring
	0,074	0,267	0 Siegelring -> Socken
	1,481	1,341	0 Socken -> Stirnband
	1,593	1,421	0 Stirnband -> Sweatshirt
	0,370	0,792	0 Sweatshirt -> suchen
	0,741	0,984	0 suchen -> Schalter
	0,370	0,742	0 Schalter -> Stempel
	1,148	1,231	1 Stempel -> Stift
	0,111	0,320	0 Stift -> Signal
	1,481	1,602	0 Signal -> Stärke
	0,296	0,669	1 Stärke -> stumpf
	0,185	0,622	0 stumpf -> sorgen
	0,259	0,712	0 sorgen -> stinken
VF29			
	0,741	1,059	0 Sonne -> singen
	0,074	0,385	0 singen -> Siechtum
	0,074	0,267	0 Siechtum -> schwingen
	0,481	0,849	0 schwingen -> saubermachen
	0,370	0,926	0 saubermachen -> sitzen
	2,556	1,340	0 sitzen -> stehen
	0,296	0,823	0 stehen -> sabbern
	0,593	1,010	0 sabbern -> seuseln
	0,074	0,385	0 seuseln -> schwimmen
	0,815	1,241	0 schwimmen -> schön
	0,889	1,340	0 schön -> Schnee
	0,000	0,000	0 Schnee -> siebzig
	1,296	1,564	0 siebzig -> senil
	0,259	0,656	0 senil -> selten
	0,037	0,192	1 selten -> Sätze
	1,741	1,701	0 Sätze -> Sinn
	0,296	0,724	0 Sinn -> selten

	0,630	1,182	0	selten -> super
	2,111	1,450	0	super -> sehr
	0,815	1,272	0	sehr -> so
	0,593	1,152	1	so -> soll
VF30				
	2,741	1,403	0	Sauer -> Säure
	0,074	0,267	1	Säure -> Sonne
	2,630	1,363	0	Sonne -> Süden
	0,000	0,000	1	Süden -> sieben
	0,111	0,320	1	sieben -> süß
	0,037	0,192	0	süß -> sechs
	0,000	0,000	0	sechs -> Straße
	0,111	0,320	1	Straße -> Stuhl
	0,037	0,192	1	Stuhl -> staunen
	0,037	0,192	1	staunen -> Stunde
	0,148	0,456	1	Stunde -> steppen
	0,000	0,000	0	steppen -> Sache
	0,556	0,934	0	Sache -> sehen
	0,667	1,074	0	sehen -> sitzen
	0,259	0,656	0	sitzen -> suchen
	0,148	0,456	0	suchen -> Sippe
	0,222	0,577	0	Sippe -> Seuche
	0,185	0,557	0	Seuche -> schlafen
	0,519	0,975	0	schlafen -> Schnupfen
	3,370	0,688	1	Schnupfen -> schniefen
	0,037	0,192	0	schniefen -> schlau
	0,593	1,083	1	schlau -> Schlupfwinkel
	0,593	0,931	1	Schlupfwinkel -> Schlupfwespe
VF31				
	0,333	0,784	0	Streit -> sehen
	0,333	0,620	0	sehen -> speisen
	0,111	0,320	0	speisen -> sollen
	0,148	0,362	0	sollen -> schon
	0,000	0,000	0	schon -> Schirm
	0,000	0,000	0	Schirm -> Schote
	0,037	0,192	0	Schote -> Schal
	0,148	0,534	0	Schal -> Schein
	0,074	0,385	0	Schein -> Sturm
	0,074	0,267	0	Sturm -> Sache
	0,148	0,456	0	Sache -> Sieg
	0,111	0,424	0	Sieg -> starr
	0,593	1,047	1	starr -> Stau
	0,259	0,712	2	Stau -> Stahl
	0,741	0,944	2	Stahl -> Stuhl
	0,481	0,893	0	Stuhl -> Sonne
	0,259	0,656	0	Sonne -> Samt
	2,963	1,160	1	Samt -> Seide
	0,074	0,267	0	Seide -> sonders

0,630	1,149	1	sonders -> sondern
0,074	0,267	0	sondern -> sehen
3,704	0,609	0	sehen -> schauen
0,074	0,267	0	schauen -> Samen
0,222	0,641	0	Samen -> sieben
1,444	1,625	0	sieben -> Sachen
0,074	0,267	0	Sachen -> Steige
0,037	0,192	0	Steige -> Sahne
1,519	1,477	0	Sahne -> Suppe
0,222	0,641	0	Suppe -> Sole
0,074	0,385	0	Sole -> schieben
0,222	0,577	0	schieben -> scheiden
0,074	0,267	0	scheiden -> Schräge
0,222	0,506	0	Schräge -> schaben
0,037	0,192	0	schaben -> scheißen
0,000	0,000	0	scheißen -> schonen
0,370	0,884	0	schonen -> schnell
0,259	0,712	0	schnell -> chic
0,148	0,456	0	chic -> Schablone
0,111	0,424	0	Schablone -> Schopf
0,111	0,424	0	Schopf -> Sargnagel
0,000	0,000	0	Sargnagel -> Suffix
0,000	0,000	0	Suffix -> Schal
0,037	0,192	0	Schal -> Sprühflasche

VF32

0,333	0,784	0	Sonne -> Seife
0,222	0,847	0	Seife -> Sauce
0,000	0,000	0	Sauce -> sind
0,000	0,000	0	sind -> Sahne
0,222	0,641	0	Sahne -> Stiel
0,222	0,698	1	Stiel -> Strafe
0,296	0,724	1	Strafe -> Stuhl
0,852	1,231	1	Stuhl -> Strand
0,185	0,557	1	Strand -> Striemen
0,037	0,192	0	Striemen -> Schrank
0,778	1,155	0	Schrank -> Schal
0,222	0,641	0	Schal -> Schule
0,370	0,792	0	Schule -> Schiff
0,185	0,557	2	Schiff -> Schaf
0,074	0,385	0	Schaf -> Schiene
0,000	0,000	1	Schiene -> Schimmel
0,185	0,483	1	Schimmel -> schinden
0,074	0,267	0	schinden -> suhlen
0,000	0,000	1	suhlen -> Summe
0,074	0,267	0	Summe -> sinken
0,148	0,456	1	sinken -> singen
0,000	0,000	0	singen -> Siegel
0,037	0,192	1	Siegel -> Sieb
0,815	1,145	1	Sieb -> sickern

	0,222	0,641	0 sickern -> Salbe
	0,148	0,456	1 Salbe -> Samen
	0,037	0,192	0 Samen -> Segel
	0,519	0,975	0 Segel -> Seide
	0,037	0,192	0 Seide -> sollen
	0,333	0,734	0 sollen -> spielen
	0,333	0,620	1 spielen -> sputen
	0,259	0,594	1 sputen -> sparen
	0,111	0,320	1 sparen -> spiegeln
	0,185	0,557	1 spiegeln -> springen
	0,111	0,424	1 springen -> Spange
	0,148	0,456	1 Spange -> Sprung
	0,111	0,424	0 Sprung -> summen
	0,000	0,000	0 summen -> Saft
	0,000	0,000	0 Saft -> schraffieren
VF33			
	2,630	1,214	0 schlafen -> Sofa
	0,111	0,424	0 Sofa -> saugen
	0,852	1,199	0 saugen -> süß
	0,370	0,792	0 süß -> Schnecke
	0,037	0,192	0 Schnecke -> sieben
	0,000	0,000	0 sieben -> Sakko
	0,333	0,784	0 Sakko -> Sofa
	0,037	0,192	1 Sofa -> Sonnenblume
	0,037	0,192	0 Sonnenblume -> sagen
	0,000	0,000	0 sagen -> Sauce
	0,185	0,622	0 Sauce -> super
	0,222	0,641	0 super -> schräg
	1,889	1,450	0 schräg -> schrullig
	0,074	0,385	0 schrullig -> Schokolade
	0,037	0,192	0 Schokolade -> Sense
	0,370	0,839	0 Sense -> Samen
	1,444	1,553	1 Samen -> Salat
	0,037	0,192	0 Salat -> Sehne
	0,000	0,000	2 Sehne -> Sahne
	0,333	0,784	0 Sahne -> schön
	0,222	0,506	0 schön -> Scham
	0,296	0,669	0 Scham -> Sonne
VF34			
	1,926	1,567	0 Sonne -> See
	0,407	1,010	0 See -> Suche
	0,370	0,688	0 Suche -> Socke
	0,037	0,192	0 Socke -> Schwur
	0,074	0,385	1 Schwur -> Schwein
	1,630	1,644	0 Schwein -> Stachel
	0,111	0,424	0 Stachel -> Sirup
	1,185	1,331	0 Sirup -> Sahne
	0,074	0,385	0 Sahne -> Stuhl

1,704	1,382	0	Stuhl -> Schrank
0,815	1,241	0	Schrank -> Steckdose
0,259	0,656	0	Steckdose -> Säule
0,185	0,396	0	Säule -> Sache
0,333	0,832	0	Sache -> Sieg
0,407	0,888	0	Sieg -> Sorge
0,222	0,698	0	Sorge -> Super
0,148	0,602	0	Super -> Sprachunterricht
0,037	0,192	0	Sprachunterricht -> Strichjunge
0,148	0,456	1	Strichjunge -> Statistik
0,259	0,594	1	Statistik -> Stolperstein
0,222	0,641	0	Stolperstein -> Systematik
0,222	0,577	0	Systematik -> Schulden
0,037	0,192	0	Schulden -> Silhouette
0,148	0,362	0	Silhouette -> Seeräuber
0,111	0,424	0	Seeräuber -> Spardose
0,111	0,424	1	Spardose -> Speck
0,000	0,000	1	Speck -> Sprung
0,000	0,000	1	Sprung -> Spruch
0,148	0,456	1	Spruch -> Spaziergang
0,000	0,000	1	Spaziergang -> Spiegelei
0,000	0,000	1	Spiegelei -> Spielregel
0,037	0,192	1	Spielregel -> Spaghetti
0,074	0,385	0	Spaghetti -> Stoff
0,148	0,456	1	Stoff -> Strategie
0,444	0,892	1	Strategie -> Stetigkeit
2,296	1,540	1	Stetigkeit -> Stabilität

VF35

0,444	1,050	0	Schlange -> Stern
3,111	1,155	0	Stern -> Sonne
0,259	0,594	0	Sonne -> Sesam
0,074	0,267	0	Sesam -> Subkultur
0,704	1,068	0	Subkultur -> Straße
0,111	0,424	0	Straße -> Segel
0,037	0,192	0	Segel -> Schnecke
0,000	0,000	0	Schnecke -> Schwedeneis
0,630	0,967	0	Schwedeneis -> Schnee
0,148	0,456	0	Schnee -> Stuhl
0,074	0,385	0	Stuhl -> Solarenergie
0,148	0,362	0	Solarenergie -> Schnellstraße
0,185	0,786	0	Schnellstraße -> schön
0,667	1,074	0	schön -> satt
0,407	0,971	0	satt -> Sinn
0,037	0,192	0	Sinn -> Samen
1,370	1,334	1	Samen -> Salat
0,148	0,456	0	Salat -> Suche
1,222	1,155	0	Suche -> sehen
0,333	0,784	0	sehen -> schneiden
0,370	0,742	0	schneiden -> Sisal

	0,000	0,000	0 Sisal -> Schmarrn
	0,593	1,118	1 Schmarrn -> Schmand
	0,000	0,000	0 Schmand -> schnell
VF36			
	0,074	0,385	0 Stuhl -> Sieb
	0,037	0,192	0 Sieb -> Stern
	0,074	0,267	0 Stern -> Sumpf
	0,037	0,192	0 Sumpf -> Schlaf
	0,074	0,267	0 Schlaf -> Sau
	3,889	0,320	0 Sau -> Schwein
	0,519	1,014	0 Schwein -> Schäfer
	0,185	0,681	0 Schäfer -> Säugling
	1,741	1,723	0 Säugling -> Saugglocke
	0,000	0,000	0 Saugglocke -> Säbelzahntiger
	0,000	0,000	0 Säbelzahntiger -> Stadt
	0,148	0,456	0 Stadt -> Satan
	0,074	0,267	0 Satan -> Schuh
	0,259	0,594	0 Schuh -> Säufer
	0,778	1,013	0 Säufer -> Sarg
	0,074	0,267	0 Sarg -> Sonne
	0,000	0,000	0 Sonne -> Stufe
	0,185	0,396	0 Stufe -> Salamander
	0,000	0,000	0 Salamander -> Socke
	0,148	0,456	0 Socke -> Ständer
	0,259	0,656	0 Ständer -> Sofa
	0,074	0,267	0 Sofa -> Schnürsenkel
	0,000	0,000	0 Schnürsenkel -> Siebbeinzelle
VF37			
	0,556	0,934	0 Stadt -> Symbol
	0,259	0,526	0 Symbol -> Serie
	0,037	0,192	0 Serie -> Saft
	0,296	0,609	0 Saft -> Stiel
	0,148	0,534	1 Stiel -> Stand
	0,407	0,844	1 Stand -> Stiftung
	0,407	0,844	1 Stiftung -> streng
	0,593	1,047	0 streng -> Soll
	0,370	0,839	0 Soll -> sind
	0,074	0,385	0 sind -> See
	0,407	1,083	0 See -> super
	0,185	0,786	0 super -> Schornstein
	1,370	1,334	0 Schornstein -> Schiff
	0,074	0,267	0 Schiff -> Schärpe
	0,370	0,742	0 Schärpe -> Scheitel
	0,148	0,602	0 Scheitel -> Sport
	0,111	0,424	1 Sport -> Spast
	0,074	0,385	1 Spast -> Spiel
	0,037	0,192	1 Spiel -> Spange
	0,111	0,320	3 Spange -> Stange

	0,037	0,192	0 Stange -> Sarg
	0,593	1,118	1 Sarg -> Saal
	0,074	0,385	1 Saal -> Sand
	0,185	0,557	0 Sand -> Sirene
	0,296	0,724	0 Sirene -> Sarg
	0,444	0,801	0 Sarg -> Spannung
	0,222	0,698	0 Spannung -> Single
	0,074	0,267	0 Single -> setzen
	0,222	0,506	0 setzen -> singen
	1,704	1,235	0 singen -> sagen
	0,185	0,622	0 sagen -> springen
	0,074	0,385	0 springen -> schlafen
	0,148	0,456	1 schlafen -> schlagen
VF38			
	2,259	1,534	0 Sauce -> Suppe
	0,000	0,000	1 Suppe -> Supraleiter
	0,037	0,192	0 Supraleiter -> Semikolon
	0,000	0,000	0 Semikolon -> Samen
	0,556	0,847	0 Samen -> Singvogel
	0,148	0,602	0 Singvogel -> Sekundenkleber
	0,111	0,320	1 Sekundenkleber -> Selter
	2,074	1,385	0 Selter -> Saft
	0,222	0,698	0 Saft -> Schwein
	1,074	1,174	0 Schwein -> Schnecke
	0,259	0,656	0 Schnecke -> Schubkarre
	0,259	0,859	0 Schubkarre -> Stelze
	0,111	0,577	0 Stelze -> Sandpapier
	0,111	0,424	0 Sandpapier -> Symmetrie
	0,037	0,192	0 Symmetrie -> Säbelzahntiger
	0,185	0,622	0 Säbelzahntiger -> Sumpf
	1,741	1,631	0 Sumpf -> Schildkröte
	0,185	0,557	0 Schildkröte -> Sieg
	0,741	1,095	0 Sieg -> Schande
	0,259	0,712	1 Schande -> Schein
VF39			
	0,037	0,192	1 Schaf -> schade
	0,037	0,192	0 schade -> Schlauch
	0,222	0,506	3 Schlauch -> Strauch
	0,000	0,000	0 Strauch -> Schieber
	0,370	0,967	0 Schieber -> Schuljunge
	0,259	0,594	1 Schuljunge -> Schuster
	0,037	0,192	0 Schuster -> Schlumpf
	0,000	0,000	0 Schlumpf -> Schach
	0,222	0,641	1 Schach -> Schaubühne
	0,074	0,385	0 Schaubühne -> Schokolade
	0,222	0,506	0 Schokolade -> Süden
	1,074	1,299	0 Süden -> Schifffahrt
	0,259	0,656	0 Schifffahrt -> sauber

	0,037	0,192	0 sauber -> schade
	0,185	0,483	0 schade -> Schund
	0,037	0,192	0 Schund -> Schlaf
	0,852	1,199	0 Schlaf -> Sand
	0,481	0,893	1 Sand -> sauber
	0,111	0,320	1 sauber -> Sack
	0,481	1,014	0 Sack -> Strolch
	0,444	1,050	0 Strolch -> Seil
	0,074	0,267	0 Seil -> Sole
	0,074	0,385	0 Sole -> sachte
VF40			
	0,556	0,934	0 Schwanz -> Seil
	0,111	0,424	1 Seil -> Seife
	0,222	0,641	0 Seife -> Serie
	0,074	0,267	0 Serie -> Seide
	0,370	0,967	1 Seide -> Saal
	0,407	0,844	1 Saal -> sauber
	0,926	1,269	0 sauber -> seriös
	0,556	0,974	0 seriös -> schön
	0,704	1,103	0 schön -> Sofa
	3,370	0,792	0 Sofa -> Sessel
	0,148	0,456	0 Sessel -> schlank
	2,111	1,502	0 schlank -> sexy
	0,148	0,602	0 sexy -> Sprudel
	0,185	0,622	0 Sprudel -> super
	0,519	1,221	0 super -> Sahne
	0,074	0,267	0 Sahne -> Stuhl
	0,037	0,192	0 Stuhl -> selten
	0,074	0,267	0 selten -> Seife
	0,481	0,935	0 Seife -> Schrank
	0,000	0,000	0 Schrank -> Stimmung
	0,074	0,385	0 Stimmung -> Schreibstift
VF41			
	0,852	1,406	0 schneiden -> Schale
	0,148	0,534	0 Schale -> Seil
	0,037	0,192	1 Seil -> Salamander
	0,074	0,267	1 Salamander -> Salz
	0,148	0,602	0 Salz -> Schere
	0,148	0,602	0 Schere -> Schatz
	0,185	0,557	0 Schatz -> Schonung
	0,259	0,656	0 Schonung -> Schule
	0,185	0,396	0 Schule -> Schal
	0,111	0,320	1 Schal -> Schande
	0,111	0,320	0 Schande -> Schiff
	1,074	1,269	0 Schiff -> Scholle
	0,074	0,267	0 Scholle -> Schein
	0,111	0,320	0 Schein -> Schelle
	0,778	1,155	0 Schelle -> Spiel

	0,407	0,747	1 Spiel -> Spitze
	0,333	0,832	1 Spitze -> Spange
	0,074	0,385	1 Spange -> sprengen
	0,037	0,192	1 sprengen -> Spaghetti
	0,185	0,786	0 Spaghetti -> Satz
	1,148	1,562	0 Satz -> setzen
	0,519	1,189	1 setzen -> Senke
	0,037	0,192	1 Senke -> sengen
	0,000	0,000	2 sengen -> singen
	0,778	1,121	0 singen -> Sekt
	0,296	0,775	0 Sekt -> Schuhe
	0,037	0,192	0 Schuhe -> Spritze
VF42			
	0,037	0,192	1 Satz -> Salz
	0,259	0,712	1 Salz -> Seife
	0,037	0,192	0 Seife -> sieben
	0,148	0,770	0 sieben -> Süden
	0,037	0,192	0 Süden -> sauber
	0,444	0,801	0 sauber -> sehen
	0,222	0,577	1 sehen -> sehr
	0,148	0,602	0 sehr -> Sache
	0,037	0,192	0 Sache -> siechen
	0,000	0,000	0 siechen -> Senf
	0,077	0,272	0 Senf -> so
	0,148	0,456	1 so -> Sonntag
	0,519	0,893	0 Sonntag -> sitzen
	1,074	1,107	0 sitzen -> Stein
	0,259	0,813	1 Stein -> Stiel
	0,074	0,267	1 Stiel -> Stiefel
	0,037	0,192	1 Stiefel -> Stern
	0,037	0,192	1 Stern -> Strumpf
	0,037	0,192	1 Strumpf -> Streichholz
	0,370	0,926	1 Streichholz -> Ständer
	0,037	0,192	1 Ständer -> Stimme
	0,148	0,602	1 Stimme -> Steuer
	0,037	0,192	1 Steuer -> Strauch
	0,148	0,456	1 Strauch -> strecken
	0,370	1,006	0 strecken -> Saft
	0,037	0,192	0 Saft -> Schirm
	0,407	0,747	0 Schirm -> Schädel
	0,111	0,424	0 Schädel -> schleifen
	1,333	1,074	0 schleifen -> schmieren
	0,185	0,622	0 schmieren -> Stecker
	0,185	0,622	0 Stecker -> sachte
	0,296	0,823	1 sachte -> sagen
	0,222	0,577	0 sagen -> Stirn
	0,000	0,000	1 Stirn -> Stängel
	0,259	0,712	1 Stängel -> stumpf
	0,111	0,424	1 stumpf -> Stand

0,407	0,844	0	Stand -> suchen
0,185	0,622	0	suchen -> singen
